# Repetitive Transcranial Magnetic Stimulation Induces Cognitive Recovery in Alzheimer's Disease via GABAergic Neuron Activation of the Cx3cl1‐Cx3cr1 Axis

**DOI:** 10.1111/cpr.70061

**Published:** 2025-05-25

**Authors:** Yunxiao Kang, Jilun Liu, Yu Wang, Jiaying Wang, Jinyang Wang, Chenming Zhou, Rui Cui, Tianyun Zhang

**Affiliations:** ^1^ Laboratory of Neurobiology Hebei Medical University Shijiazhuang China; ^2^ Neuroscience Research Center Hebei Medical University Shijiazhuang China; ^3^ Department of Oral and Maxillofacial Surgery The Second Hospital of Hebei Medical University Shijiazhuang China; ^4^ Department of Neurology The Third Hospital of Hebei Medical University Shijiazhuang China; ^5^ Core Facilities and Centers Hebei Medical University Shijiazhuang China; ^6^ Department of Human Anatomy Hebei Medical University Shijiazhuang China; ^7^ The Key Laboratory of Neural and Vascular Biology, Ministry of Education, Center for Brain Science and Disease Hebei Medical University Shijiazhuang China; ^8^ Key Laboratory of Vascular Biology of Hebei Province Hebei Medical University Shijiazhuang China

**Keywords:** Alzheimer's disease, cognitive function, Cx3cl1‐Cx3cr1 axis, GABAergic neurons, microglia, rTMS

## Abstract

This study aimed to investigate the impact of repetitive transcranial magnetic stimulation (rTMS) on cognitive recovery in Alzheimer's disease (AD) by exploring the role of GABAergic neuron activation and modulation of the Cx3cl1‐Cx3cr1 signalling axis. The 5xFAD mouse model was utilised for scRNA‐seq analysis to examine changes in gene expression post‐rTMS. Microglial phagocytic activity, amyloid plaque burden, cell–cell communication, microglial morphology and neuroinflammation markers were assessed. Following rTMS, upregulation of Cx3cl1 in GABAergic neurons was observed, leading to enhanced microglial phagocytosis, reduced amyloid plaque burden, improved cell–cell communication, altered microglial morphology and decreased neuroinflammation markers. This study demonstrates that rTMS promotes Aβ clearance and cognitive recovery in AD by activating GABAergic neurons and enhancing Cx3cl1‐Cx3cr1 signalling, providing a novel molecular target for non‐invasive AD therapy. These findings support the transition from invasive to non‐invasive AD treatments, improving patient adherence and therapeutic outcomes. Furthermore, the elucidation of cellular and molecular mechanisms facilitates drug development targeting the Cx3cl1‐Cx3cr1 axis, offering new opportunities for AD intervention.

## Graphical abstract

Schematic diagram illustrating the molecular mechanisms of the Cx3cl1‐Cx3cr1 axis mediated by rTMS activation of GABAergic neurons in the process of cognitive function recovery in AD.

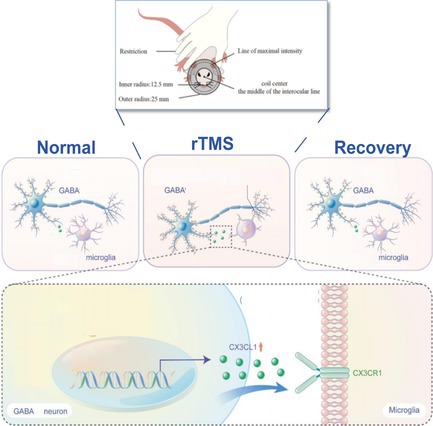

## 1 Introduction

Alzheimer's disease (AD) is a progressive neurological disorder characterised by cognitive impairment, memory loss and behavioural issues [1, 2, 3]. This disease significantly impacts patients' quality of life and social functioning, imposing a substantial burden on caregivers and healthcare systems [4, 5]. Current pharmacological treatments offer limited efficacy and are often associated with significant side effects [6, 7, 8]. Given these challenges, there is a pressing need to explore more effective and safer therapeutic approaches for AD.

In recent years, repetitive transcranial magnetic stimulation (rTMS) has garnered increasing attention as a non‐invasive neuroregulation technique [9, 10, 11]. By delivering magnetic pulses to targeted brain regions, rTMS modulates neuronal excitability, synaptic plasticity and neurotransmitter release, potentially influencing disease progression [12, 13, 14]. Compared to traditional drug therapies, rTMS is characterised by its non‐invasiveness, safety and high adjustability, making it a promising potential treatment for AD [15]. In clinical practice, rTMS has been used in the treatment of conditions such as depression and anxiety disorders, showing promising therapeutic efficacy [16, 17]. Studies have shown that rTMS has been confirmed in clinical research to improve cognitive function, psychiatric symptoms and other clinical manifestations in AD patients. However, the pathological mechanisms of AD and the mechanisms by which rTMS treats AD are still unclear. Future large‐scale, multi‐centre, randomised controlled trials are needed to explore the key mechanisms of rTMS in AD treatment and to further identify the optimal stimulation patterns and parameters of rTMS. This will provide greater help in treating AD patients and improving their quality of life [18, 19, 20, 21]. Through precise modulation of the brain using rTMS technology, extensive research and application could pave the way for new possibilities and prospects in treating AD and other neurological disorders.

GABAergic neurons, a key inhibitory neuronal subtype in the central nervous system, play a critical role in regulating neuronal activity [22, 23, 24]. Dysfunction in GABAergic signalling has been implicated in the pathogenesis of neurodegenerative diseases, including AD [25, 26]. GABAergic neurons are a major type of inhibitory neurons in the central nervous system, and their regulation of neuronal activity is crucial. Studies suggest that dysfunction of GABAergic neurons may be related to the pathogenesis of neurodegenerative diseases such as AD [22, 23, 24]. GABA is one of the two inhibitory neurotransmitters in the CNS. Common markers for GABAergic neurons include GAT1, GABBR2, GAD65 and GAD67. CX3CL1 (fractalkine)‐CX3CR1 signalling represents one of the most important communication pathways between neurons and microglia. The expression of CX3CL1 in neurons and its receptor CX3CR1 in microglia determines specific interactions, playing an important role in the maturation and functional regulation of these cells [27]. Therefore, exploring how rTMS can activate GABAergic neurons to modulate the Cx3cl1‐Cx3cr1 axis in the brain holds promise as an important direction for developing new AD treatment strategies.

This study utilised the 5xFAD mouse model to simulate AD and explore the therapeutic effects of rTMS. The research methodology involved analysing mouse brain tissues before and after rTMS treatment using single‐cell RNA sequencing (scRNA‐seq) technology to unveil cell‐type‐specific responses [28, 29, 30]. Ligand–receptor (L–R pairs) interaction analysis was conducted to investigate the impact of rTMS on brain signalling pathways [31, 32]. Additionally, amyloid plaque deposition was monitored using a multiphoton microscope, and changes in neuroinflammation markers, cell morphology features and phagocytic capacity were evaluated through immunohistochemistry, flow cytometry and Western blotting methods [33, 34]. Finally, bioinformatics analysis integrating transcriptomic and single‐cell data was performed to screen and identify key signalling molecules and pathways [35, 36].

Through this research, our aim is to elucidate the role of rTMS‐mediated activation of GABAergic neurons in the Cx3cl1‐Cx3cr1 axis in the restoration of cognitive function in AD. Further understanding the impact of rTMS on cellular communication and signal transduction may provide a scientific basis for identifying new molecular targets and signalling pathways discovered in the study, thus driving innovative developments in AD treatment. The research findings could potentially offer more effective treatment options for AD patients and open up new research directions in the field of neuroscience, bearing significant clinical and societal implications.

## 2 Materials and Methods

### 2.1 Ethics Statement

All mouse experiments in this study were conducted in accordance with international and domestic ethical guidelines and regulations regarding the use of experimental animals. The Animal Ethics Committee of Hebei Medical University approved the experimental procedures (Approval Number: IACUC‐Hebmu‐2023054). Throughout the study, all experimental animals were treated with respect and humane care. Housing and handling conditions were designed to minimise pain and stress. At the conclusion of the experiments, the mice were humanely euthanized. Experimental animals were provided by Beijing Vital River Laboratory Animal Technology Co. Ltd. and Jackson Laboratory. Additionally, strict adherence to research ethics principles was ensured, with full respect for all individuals involved in the study.

### 2.2 Experimental Animals

5xFAD transgenic mice were obtained from Jackson Laboratory (006554, USA). These transgenic mice carried five mutations in the familial AD amyloid precursor protein (APP) and presenilin 1 (PSEN1) genes, leading to a rapid increase in brain amyloid deposits under the regulation of the neuron‐specific Thy1 promoter. Age‐matched, non‐transgenic C57Bl/6 (NTG) mice were obtained from Vital River Laboratory Animal Technology Co. Ltd. (Beijing, China). Homozygous GAD‐Cre mice (Gad2tm2(cre) Zjh/; 010802, Jackson Laboratory, USA) were crossbred with heterozygous 5xFAD (AD) mice to generate AD‐GAD‐Cre and NTG‐GAD‐Cre offspring, which were used in this study. All mice were male, 4 months old, and housed under controlled environmental conditions (temperature: 23°C ± 2°C, relative humidity: 50% ± 10%) with ad libitum access to food and water [37, 38, 39]. To assess changes in social behaviour across groups of mice, we conducted behavioural testing. The order of behavioural tests was the three‐chamber social interaction test, open field test, Y‐maze alternation test, and Morris water maze test. Each experimental group consisted of six mice. The three‐chamber social interaction test and open field test were performed on Day 22, followed by the Y‐maze alternation test and Morris water maze test, which were performed on alternate days after allowing the mice to recover.

### 2.3 rTMS

rTMS treatment was performed using a magnetic stimulator (CCY‐II, Wuhan Irid Medical Equipment, China). To minimise novelty effects, all mice underwent three consecutive days of restraint and rTMS coil adaptation prior to treatment. The 5xFAD mice were placed in a custom‐made restraining device, which temporarily restricted movement while keeping the head exposed. During rTMS stimulation, the mice exhibited normal breathing patterns without signs of distress. A circular coil (diameter: 6.5 cm) was positioned above the mice's heads at the coil's centre. rTMS was delivered at a fixed daily time for 14 consecutive days, consisting of 20 bursts of 20 Hz stimulation per session at a magnetic stimulation intensity of 138% RMT, with a 1‐s interval between bursts. Following the 14‐day treatment period, the mice were allowed a 7‐day recovery period [40].

### 2.4 Stereotaxic Adeno‐Associated Virus (AAV) Infusion and Drug Administration

AD‐GAD‐Cre and NTG‐GAD‐Cre mice were anaesthetised with 4% isoflurane and placed on a stereotaxic frame. Once secured in the apparatus, the isoflurane concentration was reduced to 1.5% and administered intranasally during the surgery. Following scalp disinfection, the skin was incised along the midline. The left prefrontal cortex was injected with the virus at coordinates AP +1, ML +0.5, DV −0.9, at a rate of 0.1 μL/min. A virus solution containing 1.5 μL GPAAV‐EF1a‐DIO‐hChR2(H134R)‐mCherry (1 × 10^13^ viral particles/ml, GM‐4917AA8E2‐200, Jinman Biotechnology (Shanghai) Co. Ltd., China) or a control vector AAV5‐EF1a‐DIO‐mCherry without ChR2 (1 × 10^13^ viral particles/ml, Jinman Biotechnology (Shanghai) Co. Ltd., China) was injected using a 1 mL Hamilton syringe. After the injection, the skin was sutured, and the mice were kept on a warm mat without anaesthesia until they regained consciousness and mobility. A minimum resting period of 3 weeks was allowed for all mice before initiating any experimental procedures to ensure proper recovery and virus expression. Virus expression was confirmed in all mice. A fibre optic cannula (Doric Lenses) was placed and secured at the site of virus expression. The cannula was carefully mounted on the cortex. The main groupings were as follows: (1) NTG‐ChR2, AD‐ChR2 and AD‐ChR2 + rTMS [39]. Each experimental group consisted of six mice.

Minocycline hydrochloride (Mino) (Cat# M9511, Sigma‐Aldrich) was dissolved in water (10 mg/mL) and administered at a dose of 50 mg/kg. For intracranial microinfusion, a guide cannula (inner diameter 0.35 mm, RWD) was implanted at least 2 weeks before behavioural testing to allow sufficient recovery time. Mino diluted in artificial cerebrospinal fluid (ACSF) to a concentration of 40 mM was injected unilaterally through the guide cannula at a depth of 0.2 mm below the guide, with local application of ACSF (250 nL) as a control. The CX3CR1 antagonist JMS‐17‐2 (Cat# HY‐123918, MedChemExpress) was dissolved in corn oil (10 mg/mL) and administered at a dose of 10 mg/kg via intraperitoneal injection. JMS‐17‐2 diluted in corn oil to a concentration of 10 mM was microinjected into the left prefrontal cortex, with local application of the vehicle solution (corn oil, 150 nL) as a control. The main groupings were as follows: (1) AD‐rTMS + Saline and AD‐rTMS + Mino; (2) AD + rTMS + Vehicle and AD + rTMS + JMS‐17‐2 [41]. Each experimental group consisted of six mice.

For virus injection, the skull was exposed, and holes were drilled (diameter 0.5 mm; anterior–posterior (AP) 0.85, medial–lateral (ML) ±0.5, dorsal–ventral (DV) −4.6; AP 0.7, ML ±0.75, DV −4.9; AP 0.6, ML ±0.75, DV −4.9 mm). A total of six injection sites were used. Cre‐inducible adeno‐associated virus serotype 9 (AAV9), containing ChR2 and enhanced yellow fluorescent protein (EYFP) fusion constructs [AAV‐hSyn‐DIO‐hChR2(H134R)‐EYFP, ≥ 1 × 10^13^ vg/mL, Addgene, MA], was injected to drive Cre‐dependent co‐expression of ChR2 and EYFP. The injection needle was slowly lowered to the HDB and held in place for 1 min. A 0.125 μL injection of virus was given at each site within 3 min, followed by a 1‐min incubation before the needle was slowly removed. After 3–4 weeks, ChR2 expression in the olfactory bulb was confirmed, and in vitro whole‐cell recordings were performed [42].

### 2.5 Acquisition of Single‐Cell Transcriptome Data

A single mouse brain tissue from 5xFAD and rTMS models, respectively, was selected for scRNA‐seq analysis on Day 21. The brain cell suspension was classified using the LSRFortessa cell analyser (BD Biosciences, CA, USA). RNA was purified, and libraries were prepared following the instructions of the 10× v2 5′ Expression Library Prep Kit. Base calling was performed using RTA2 software, followed by alignment to the GRCm38 reference genome provided by 10X Genomics using Cellranger mkfastq (v3.0.2). The expression matrix generated by Cellranger was then loaded into the Seurat (v4.0.2) R package for downstream analysis, including t‐distributed stochastic neighbour embedding (t‐SNE) analysis [43].

### 2.6 Single‐Cell Analysis

Data quality control was conducted based on the criteria nFeature_RNA > 200, nCount_RNA < 50,000 and percent.mt < 15. To reduce the dimensionality of the scRNA‐Seq dataset, principal component analysis (PCA) was performed using the top 2000 highly variable genes based on variance. The Elbowplot function of the Seurat package was utilised to select the top 17 principal components (PCs) for downstream analysis. The clustering of major cell subpopulations was identified using the FindClusters function of Seurat with a default resolution (res = 0.5). Subsequently, a non‐linear dimensionality reduction of scRNA‐seq sequencing data was performed using the t‐SNE algorithm. Markers of various cell subpopulations were identified using the Seurat package, and cell annotation was carried out using the online resource CellMarker. Temporal analysis was conducted using the “monocle” package [44], followed by enrichment analysis using the “ClusterGvis” package.

### 2.7 Transcriptome Sequencing

Mouse brain cortex tissues from the AD‐ChR2 group and AD‐ChR2 + rTMS group were selected, and 20–50 mg of tissue was sliced on dry ice. Total RNA was extracted from the tissues using TRIzol (Cat. No. 15596026, Thermo Fisher, USA), and the purity and concentration of the extracted RNA were assessed using the Nanodrop 2000 UV–Vis spectrophotometer (Thermo Fisher, USA). Following the instructions of the PrimeScript RT reagent Kit (RR047A, Takara, Japan), RNA was reverse‐transcribed into cDNA for transcriptome sequencing. Differential analysis was performed using the “limma” package in R language, with |log2(Fold Change)| > 1 and a significant *p* value < 0.05 as the criteria for selecting differentially expressed genes, leading to the identification of differentially expressed genes [45].

### 2.8 Functional Enrichment Analysis of Candidate Targets

For functional enrichment analysis of the gene set, online analytical tools, the DAVID database (https://david.ncifcrf.gov/home.jsp), and gene GO annotations from the org.Hs.eg.db R package (version 3.1.0) were utilised as the background. The genes were mapped to the background set, and enrichment analysis was conducted using the clusterProfiler R package (version 3.14.3) to obtain enriched gene set results. The criteria were set with a minimum gene set of five, a maximum gene set of 5000, *p* value < 0.05, and FDR < 0.1 [46].

### 2.9 Machine Learning Algorithm

The random forest (RF) machine‐learning algorithm was employed to further refine feature gene selection using the randomForest R package. The number of trees (ntrees) and the number of variables randomly sampled at each split (mtry) were set to 500 and 3, respectively. Initially, the optimal number of trees was determined by minimising cross‐validation error. Subsequently, a random forest model was constructed using this optimised tree count. To identify highly significant feature genes, the importance score of each gene was computed. Genes with an importance score greater than 2 were selected as candidate feature genes [47].

### 2.10 Preliminary Testing in Experimental Mice

Prior to behavioural testing, each mouse underwent habituation training in the testing apparatus on the day before the experiment. To ensure proper adaptation, the mice were allowed to freely explore the experimental environment for at least 1 h before the formal testing phase. The preliminary test was conducted in a square testing chamber divided into four equal sections, with each block measuring 50 cm × 50 cm × 40 cm^3^. The chamber floor was marked with boundary lines to delineate the areas. Mice were placed in the chamber and given 30 s to acclimate before the test. Each mouse then underwent a 10‐min test session, during which their movement patterns were recorded by a video camera positioned 120 cm outside the testing area. Key behavioural metrics included the time spent in the central area (defined as having all four limbs simultaneously positioned within the central square) and the total distance travelled within that zone. The testing environment was maintained under standard lighting conditions (800 lx) [48].

### 2.11 Three‐Chamber Social Behaviour Test

The three‐chamber social behaviour test was performed in a rectangular apparatus measuring 60 × 44 × 40 cm^3^, which was divided into three interconnected chambers. Glass panels were used to control access between chambers at different stages of the experiment. In the first phase (adaptation period), test mice were placed in an empty central chamber and allowed 10 min of free exploration across all compartments. In the second phase (social preference test), an unfamiliar mouse (stranger #1) from a separate cage was placed inside a metal enclosure in the left chamber, while the right chamber remained empty. The test mouse was then allowed to freely explore all compartments, and the time spent interacting with stranger #1 versus the time spent in the empty chamber was recorded over a 10‐min period. In the third phase (social novelty test), the test mouse was briefly confined in the central chamber, while a second unfamiliar mouse (stranger #2) was introduced into the previously empty right chamber. The barriers were then removed, and the test mouse was allowed to explore both social compartments freely for another 10 min. The time spent interacting with stranger #2 was recorded as an indicator of the test mouse's preference for novel social interactions [49].

### 2.12 Y‐Maze Test

The Y‐maze test was conducted in a three‐arm maze, which consisted of a central starting zone and three branching arms forming a Y‐shaped structure. Before the experiment, mice were given a habituation period to freely explore the maze and acclimate to the testing environment. Each arm of the maze measured 45 cm × 10 cm × 15 cm, and a food reward was placed at the bottom of each branch. At the beginning of each trial, mice were placed in the central start zone, ensuring a consistent starting position for all test subjects. Their behaviour in the maze, including exploration patterns, dwell time and the number of entries into each arm, was recorded using video tracking cameras. Following habituation, mice underwent a training phase to familiarise themselves with the maze layout and food reward locations. After successful training, a testing phase was conducted, during which the food placements were altered. The mice's ability to locate the correct food site based on their prior learning was assessed. Final data analysis focused on exploration time, frequency of arm entries, and the success rate in identifying the reward location, serving as indicators of spatial learning and memory function [50].

### 2.13 Morris Water Maze Experiment

A circular water reservoir with a diameter of 120 cm and a height of 50 cm was utilised for the experiment. The pool was filled with clear water maintained at a temperature similar to that of the mice (*n* = 6). A circular escape platform with a diameter of 15 cm was placed at a fixed location within the pool, submerged 2 cm below the water surface and painted an opaque colour to ensure it remained hidden from view. Prior to the experiment, mice were acclimated to the experimental environment, including the testing room and the water reservoir, to minimise stress‐induced behavioural variability. During training sessions, mice were placed at various starting points within the pool, and their swimming behaviour was recorded. The mice were initially trained to locate the escape platform with the aid of visual cues, which were subsequently removed in the testing phase to assess their spatial navigation and memory retention [51].

### 2.14 Brain Slice Preparation

Upon completion of behavioural experiments (*n* = 6), mice were anaesthetised with isoflurane, and their brain tissue was promptly extracted and immersed in ice‐cold artificial cerebrospinal fluid (ACSF) containing 250 mM sucrose, 26 mM NaHCO_3_, 10 mM glucose, 10 mM MgSO_4_, 2 mM KCl, 1.3 mM NaH_2_PO_4_ and 0.2 mM CaCl_2_. Brain slices, including those from the prefrontal cortex, were prepared in a cold‐modified ACSF using a vibratome (VT‐1200s, Leica, Germany). Subsequently, the slices were transferred to a storage chamber containing standard ACSF (126 mM NaCl, 26 mM NaHCO_3_, 10 mM glucose, 3 mM KCl, 2 mM CaCl_2_, 1.25 mM NaH_2_PO_4_ and 1 mM MgSO_4_), allowed to recover for 30 min at 34°C, followed by an additional 1‐h recovery period at room temperature (25°C ± 1°C) before commencing recording. Throughout the slice preparation process, all solutions were saturated with 95% O_2_/5% CO_2_ (volume/volume) [52].

### 2.15 Preparation of Brain Frozen Sections

Mouse brain tissue (*n* = 6) was extracted and fixed in 4% paraformaldehyde overnight at 4°C, followed by a 48‐h immersion in 30% sucrose for cryoprotection. The tissue was then placed on a cryostat and sectioned into 30 μm coronal slices of the frontal lobe, followed by rinsing in PBS three times. Upon completion of the sectioning process, the slices were used for subsequent staining studies [53].

### 2.16 Multiphoton Imaging and Data Acquisition

During the experiment, mice were anaesthetised with 1.5% isoflurane, and circular craniotomies were performed using a dental drill. An 8 mm diameter glass coverslip was placed over the viral expression area and secured to the surrounding skull with a mixture of dental cement and super glue. Texas Red dextran was intravenously injected into the retro‐orbital sinus to visualise the vasculature. One day before imaging, mice received an intraperitoneal injection of methoxy‐XO4 (10 mg/kg, B5769, Apexbio China), which binds specifically to amyloid plaques, enabling visualisation through the cranial window using a multiphoton microscope. Imaging was conducted using the FluoView FV1000MPE multiphoton laser scanning system (Olympus) mounted on a BX61WI microscope, equipped with a 25× long‐working‐distance objective (numerical aperture = 1.05). A mode‐locked titanium‐sapphire laser (MaiTai, Spectra‐Physics, Fremont, California) provided two‐photon excitation at 800 or 860 nm. Fluorescence signals were detected using three photomultiplier tubes (PMT, Hamamatsu, Japan), capturing wavelengths of 380–480 nm, 500–540 nm and 560–650 nm, respectively. While PMT settings remained constant across experiments, laser power was adjusted as needed. Pathological imaging of amyloid plaques was performed at an 800 nm excitation wavelength with 1× digital zoom. Image stacks obtained through multiphoton microscopy were analysed using ImageJ. Maximum intensity projections (MIP) were generated from each Z‐stack to quantify amyloid plaque number and load. The percentage of cortical area occupied by amyloid‐positive signals was determined via thresholding and segmentation of the projection images [39].

### 2.17 Immunofluorescence Staining

Freshly dissected mouse brains (*n* = 6) were fixed in a solution of 15% glycerol and 4% paraformaldehyde for 72 h. Coronal slices (40 μm) were sectioned using a Vibratome (Leica VT1000 S, Deer Park, IL) and stored in cryoprotectant at −20°C. For confocal imaging of microglia, antigen retrieval was performed on 40 μm coronal brain sections labelled with methoxy‐XO4 for amyloid plaques. The tissue slices were permeabilized in TBS containing 0.5% Triton X‐100, blocked with normal goat serum, and incubated overnight at 4°C with primary antibodies: anti‐Iba‐1 (1:500, ab178846, Abcam, UK), Ki67 (1:250, ab16667, Abcam, UK), Amyloid β (6E10, 1:500, 24–480, Prosci, USA), and Amyloid β (N) (82E1, 1:500, IBL 10323, Immuno‐Biological Laboratories, Minneapolis, MN). The following day, slices were incubated at room temperature for 1 h with Alexa Fluor 488‐conjugated (1:500, ab150077, Abcam, UK) or Alexa Fluor 647‐conjugated (1:500, ab150115, Abcam, UK) secondary antibodies. Finally, the sections were mounted on glass slides with Vectashield mounting medium (Vector Laboratories) without DAPI. Z‐stack images were acquired using an inverted Olympus FV3000RS confocal laser‐scanning microscope (Japan) with a 40× objective for morphological analysis. For amyloid plaque load analysis, antigen retrieval was performed on all sections using citrate buffer, followed by permeabilization in TBS with 0.5% Triton X‐100 and blocking with normal goat serum. Visualisation and imaging were conducted using a Zeiss Axiovert 123 M fluorescence microscope (Germany) with a 10× objective. The amyloid beta load in hippocampal and cortical regions was quantified by setting a positive signal threshold [39].

### 2.18 3D Reconstruction

Thirty‐micrometre‐thick tissue slices were stained with anti‐Iba‐1 antibody (ab178846, Abcam, UK) and incubated for 24 h, followed by Alexa Fluor 488‐conjugated secondary antibody staining. Imaging was performed using a Zeiss LSM880 microscope with a 40×/1.3 NA oil immersion objective, ensuring consistent imaging parameters (laser power, gain and offset) across all experiments. Image analysis was conducted using IMARIS 9.6.2 software (Bitplane) on 512 × 512‐pixel resolution images. The “filament” function in IMARIS was utilised to quantify the length and branching points of microglial processes. Each mouse was considered an independent sample. To assess microglial phagocytic capacity, 3D surface renderings of microglia were generated, with thresholds set to ensure accurate reconstruction of microglial processes. GAD65/67 spots were reconstructed using the “Spots” function. Finally, the number of GAD65/67 spots fully enclosed within microglial surfaces (distance ≤ 0 μm) was evaluated using the “Split into Surface Objects” plugin in IMARIS Matlab (MathWorks, Natick, MA, USA) [41].

### 2.19 Flow Cytometry and Aβ Phagocytosis Experiment

Mice were intraperitoneally injected with fibrillar β‐amyloid plaques labelled with methoxy‐XO4. Three hours post‐injection, mice were anaesthetised, and whole‐brain tissue was harvested and prepared into a single‐cell suspension by perfusion with 50 mL of 1× PBS. The cell suspension was centrifuged at 1200 g for 5 min to collect the cell pellets, which were subsequently resuspended in 5 mL of 30% Percoll solution and centrifuged at 1200 g for 30 min at room temperature to form a gradient. The collected cells were stained with antibodies against CD11b‐APC/Cy7 (101225), CD45‐APC (147707), CD36‐PerCP/Cy5.5 (102619), CD68‐BV711 (137029) and Colony Stimulating Factor 1 Receptor (CSF‐1R)‐PE (165203), all purchased from BioLegend (San Diego, California). Antibody staining was performed according to the manufacturer's instructions. Flow cytometry analysis was conducted using the BD Biosciences FACS FORTESSA system, and data were processed with FlowJo software version 7.6.1 (FlowJo LLC, Ashland). To establish the methoxy‐XO4 threshold for non‐phagocytic cells, wild‐type mice injected with methoxy‐XO4 were used as controls [39].

### 2.20 Western Blot

Cell lysis was carried out using enhanced RIPA lysis buffer containing protease inhibitors (P0013B, Beyotime Biotechnology Co. Ltd., Shanghai, China), followed by protein concentration determination using a BCA protein quantification kit (P0012, Beyotime Biotechnology Co. Ltd., Shanghai, China). The proteins were separated by 10% SDS‐PAGE and then transferred to a PVDF membrane. Blocking with 5% BSA at room temperature for 2 h to prevent nonspecific binding, the membrane was then incubated with primary rabbit antibodies against Cx3cl1 (ab25088, dilution ratio 1:1000, Abcam, USA), TNF‐α (ab183218, dilution ratio 1:1000, Abcam, USA), IL‐1β (ab254360, dilution ratio 1:1000, Abcam, USA), IL‐6 (ab290735, dilution ratio 1:1000, Abcam, USA) and GAPDH (ab9485, dilution ratio 1:100, Abcam, USA). Following the washing steps, the membrane was then incubated with an HRP‐conjugated secondary antibody, goat anti‐rabbit IgG (ab6721, dilution ratio 1:5000, Abcam), for 2 h. Membranes were washed with TBST for 5 min ×3 times before detection using a chemiluminescence imager. Lastly, ImageJ 1.48u software was utilised for protein quantification analysis, calculating protein levels based on the ratio of grayscale values of each protein to the internal control GAPDH [54]. Each experiment was repeated three times.

### 2.21 RT‐qPCR

Total RNA was extracted from tissues using Trizol reagent (Product number: 15596026, Invitrogen, USA). The concentration and purity of RNA were determined at 260/280 nm using a NanoDrop LITE spectrophotometer (Model: ND‐LITE‐PR, Thermo Scientific, Germany). The extracted total RNA was reverse transcribed to cDNA using the PrimeScript RT reagent Kit with gDNA Eraser (Catalogue number: RR047Q, TaKaRa, Japan). Subsequently, RT‐qPCR analysis of the genes was performed using the SYBR Green PCR Master Mix kit (Catalogue number: 4364344, Applied Biosystems, USA) and ABI PRISM 7500 sequence detection system (Applied Biosystems).

The primers for each gene were synthesised by TaKaRa (Table S1), with GAPDH serving as the reference gene. The relative expression levels of the genes were analysed using the 2^−ΔΔC^t method, where ΔΔCt = (average Ct value of the target gene in the experimental group—average Ct value of the reference gene in the experimental group)—(average Ct value of the target gene in the control group—average Ct value of the reference gene in the control group) [55, 56, 57]. All RT‐qPCR analyses were performed in triplicate.

### 2.22 Statistical Analysis

All data were processed using GraphPad Prism 8.0. Metric data were presented as mean ± standard deviation (mean ± SD). Comparison between two groups of data was conducted using an unpaired *t*‐test, and comparison among multiple groups was performed using one‐way analysis of variance (ANOVA).

The homogeneity of variance was tested using Levene's test. If the variance was homogeneous, pairwise comparisons were made using Dunnett's t‐test and LSD‐t test. If the variance was not homogeneous, Dunnett's T3 test was used. Pearson correlation analysis was utilised to assess the correlation between genes and immune cell contents [58]. A *p* value < 0.05 indicated statistical significance between the two sets of data.

## 3 Results

### 3.1 scRNA‐Seq Analysis of the Impact of rTMS on Cell Spectrum in AD Mouse Brain Tissue

**FIGURE 1:**
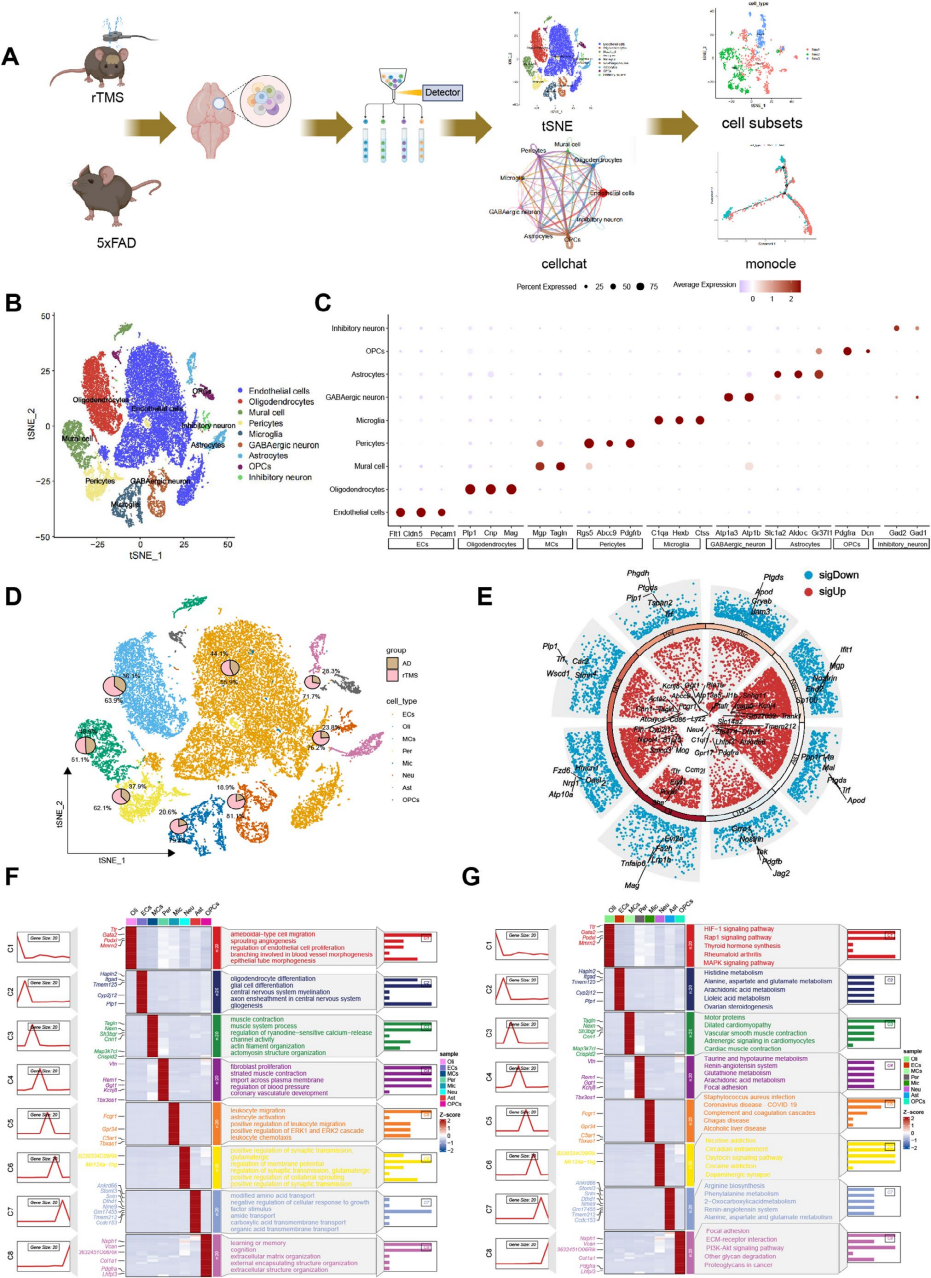
Single‐cell landscape analysis in rTMS samples based on scRNA‐seq. (A) Schematic representation of the single‐cell analysis pipeline for extracting cells from the brain tissues of 5xFAD and rTMS model mice using 10× Genomics technology; (B) t‐SNE clustering plot annotating cell types after batch effect removal; (C) Dot plot displaying specific gene expressions of major cell types, with red indicating high expression and blue indicating low expression. The size of the circles represents the percentage of cells expressing the indicated gene; (D) t‐SNE clustering plot representing the proportions of various cell types in brain tissue samples from 5xFAD and rTMS model mice; (E) Circular plot showing differentially expressed genes in cell subtypes, with red indicating high expression and blue indicating low expression; (F) Heatmap of GO‐BP enrichment analysis for each cell subtype, with the left line plot representing gene expression abundance, the middle heatmap representing gene cluster expression and the right side showing GO‐BP enrichment results; (G) Heatmap of KEGG enrichment analysis for each cell subtype, with the left line plot indicating gene expression abundance, the middle heatmap showing gene cluster expression and the right side displaying KEGG enrichment results.

rTMS has been shown to improve mental illnesses and neuropathic pain by modulating affected neural circuits [59, 60]. Nonetheless, the mechanisms and specific neural circuits through which rTMS exerts these beneficial effects remain poorly understood. In this study, to investigate the impact of rTMS on cells in the brain tissue of AD mice, we randomly selected brain tissues from a 5xFAD (AD) mouse and an rTMS model mouse for scRNA‐seq analysis. The experimental design workflow is illustrated in Figure 1A. We integrated and filtered out low‐quality cells from the data using the Seurat package (Figure S1A). Subsequently, highly variable genes were selected based on gene expression variance, and the top 2000 variable genes were chosen for downstream analysis (Figure S1B). The ElbowPlot method was employed for dimension selection in the PCA model to determine the optimal number of PCs, followed by non‐linear dimensionality reduction using t‐SNE on the top 17 PCs (Figure S1C). Through clustering analysis, we identified 19 clusters (Figure S1D) and annotated the cells using known lineage‐specific marker genes in combination with the CellMarker online database (Figure S1E). This led to the identification of eight cell categories as follows (Figure 1B): neurons (Neu), oligodendrocytes (Oli), microglia (Mic), oligodendrocyte precursor cells (OPCs), astrocytes (Ast), endothelial cells (ECs), mural cells (MCs) and pericytes. The composition of different cell types in each sample is shown in Figure S1F. Figure 1C illustrates the expression patterns of marker genes for each cell type. Furthermore, we compared the distribution of cell subtypes in AD and rTMS model mice, revealing a significant increase in the proportion of neurons, oligodendrocytes, microglia and astrocytes in the rTMS model compared with the AD group (Figure 1D). Enrichment analysis of differentially expressed genes in these eight cell classes showed involvement in various signalling pathways such as synaptic transmission, cell activation and multiple metabolic pathways (Figure 1E–G).

These findings indicate that rTMS on AD mouse brain tissue can annotate eight cell types involved in synaptic transmission, cell activation and various metabolic pathways.

### 3.2 The Impact of rTMS Stimulation on Cell–Cell Communication and Functional Neuronal Reconstruction in AD Brain Tissue

**FIGURE 2:**
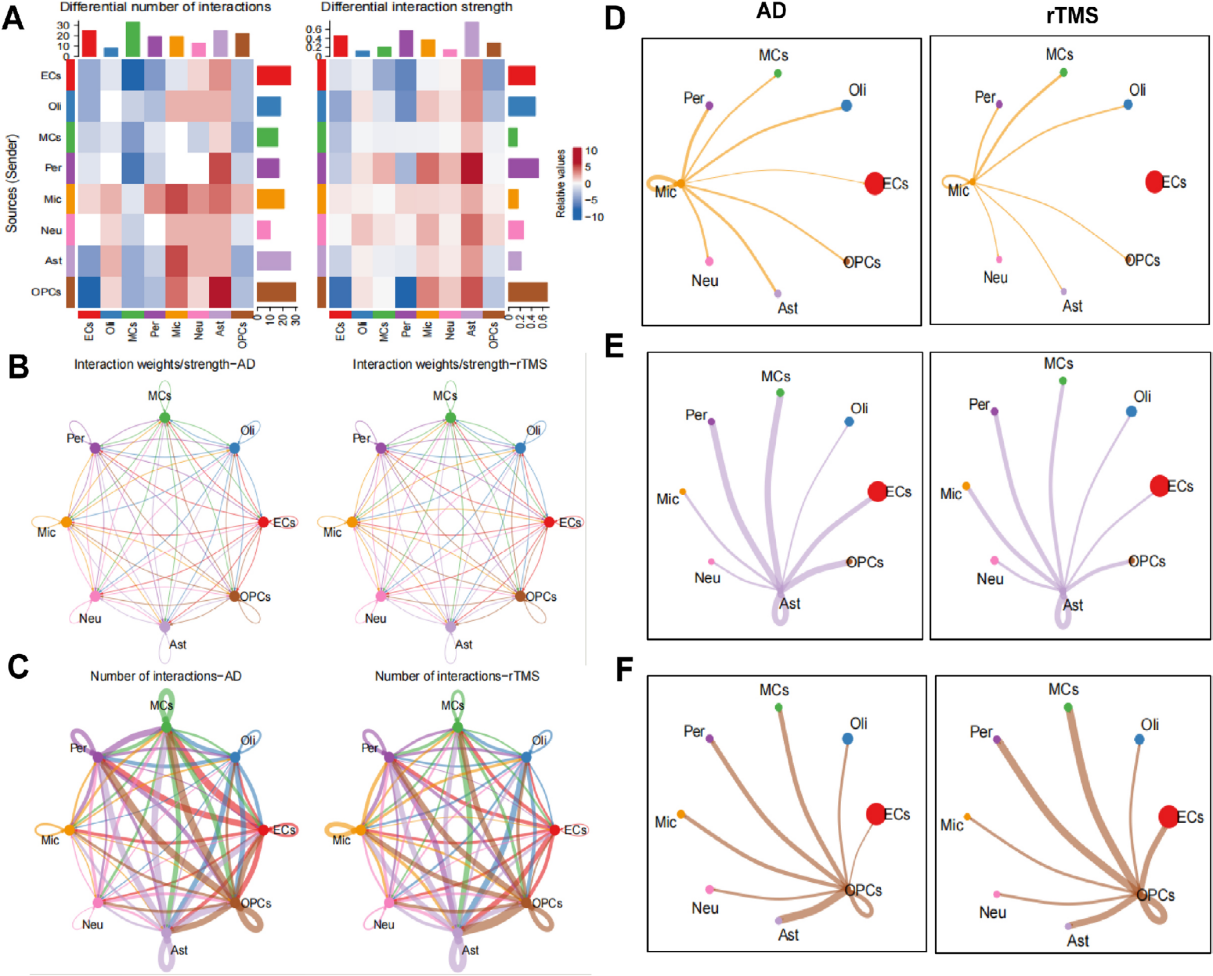
Identification of cell–cell interactions in the tissue microenvironment during AD treatment with rTMS stimulation. (A) The heat map displays differences in interaction quantity or strength. The top colour bar represents the sum of column values (inbound signals), and the right‐side colour bar represents the sum of row values (outbound signals). In the colour bar, red (or blue) signifies an increase (or decrease) in signals relative to the rTMS group compared to the AD group; (B, C) Circular plots illustrating cell communication in AD and rTMS group samples, with line thickness indicating the strength and quantity of pathway interactions; (D, F) Demonstrating the interaction scenarios of microglia, astrocytes, OPCs cells with neurons in AD and rTMS group samples.

In order to elucidate the mechanism of cellular evolution during AD treatment with rTMS stimulation, we investigated the role of ligand‐receptor interaction‐mediated cell–cell communication across all cellular components. By calculating the communication probabilities of all ligand–receptor (L–R pairs) interactions associated with each signalling pathway, we inferred the communication probabilities at the pathway level to study the influence of rTMS stimulation on the communication and interactions among cell phenotypes in AD brain tissue. Figure 2A–C illustrate the quantity of cell–cell communications and interaction strengths among eight cell subgroups. OPCs exhibited higher output interaction strength and number of interacting cells (Figure 2A), while compared to the AD group, microglia, astrocytes and neurons showed an increased number of interacting cells (Figure 2B,C).

Existing research has indicated that a characteristic of brain injury is the loss of functional neurons and activation of glial cells. In adult mice, astrocytes typically remain quiescent unless activated by injury or disease without undergoing proliferation. Apart from astrocytes, OPCs and microglia can also be activated and proliferate rapidly at the site of injury or within a diseased brain, leading to the reprogramming of reactive glial cells into functional neurons for brain repair [61]. We further conducted heterogeneous analysis on microglia, astrocytes and OPCs. The results revealed that these cell types regrouped into two distinct clusters (Figure S2A). By utilising Monocle to construct trajectories for each cell type, we observed that different cell types followed distinct trajectories. The changes in cells over pseudotime are depicted in Figure S2B, with different clusters enriching along different paths (Figure S2C).

Furthermore, we explored the interactions between microglia, astrocytes, OPCs and neurons. The results showed that in the rTMS group, there was an enhanced connection between microglia and neurons, denoted by thicker lines, while astrocytes showed no significant changes, and the interactions of OPCs were weakened (Figure 2D–F). Additionally, subgroup analysis of neurons revealed that neurons could be reclustered into three classes (Figure S3A). Monocle also demonstrated three branching points (Figure S3B), with neuronal subgroup 3 primarily located at the branching origin, gradually distributing into neuronal subgroups 1 and 2 (Figure S3C). GABAergic neurons and glutamatergic neurons are the two main neuronal types based on neurotransmitter distribution, playing crucial roles in brain signalling [22, 62]. Analysis of neuronal subgroups indicated that neuronal subgroup 1 predominantly comprised inhibitory GABAergic neurons, while neuronal subgroup 2 mainly consisted of excitatory glutamatergic neurons (Figure S3D). Furthermore, the gene expression patterns of inhibitory GABAergic neurons and excitatory glutamatergic neurons were illustrated (Figure S3E).

Overall, the results suggest that rTMS stimulation during AD treatment may mediate the interaction between microglia and neurons, thereby impacting brain repair.

### 3.3 The Effects of Optogenetic Regulation and rTMS Therapy on Cognitive Function and Inflammatory Factor Expression in AD Mice

**FIGURE 3:**
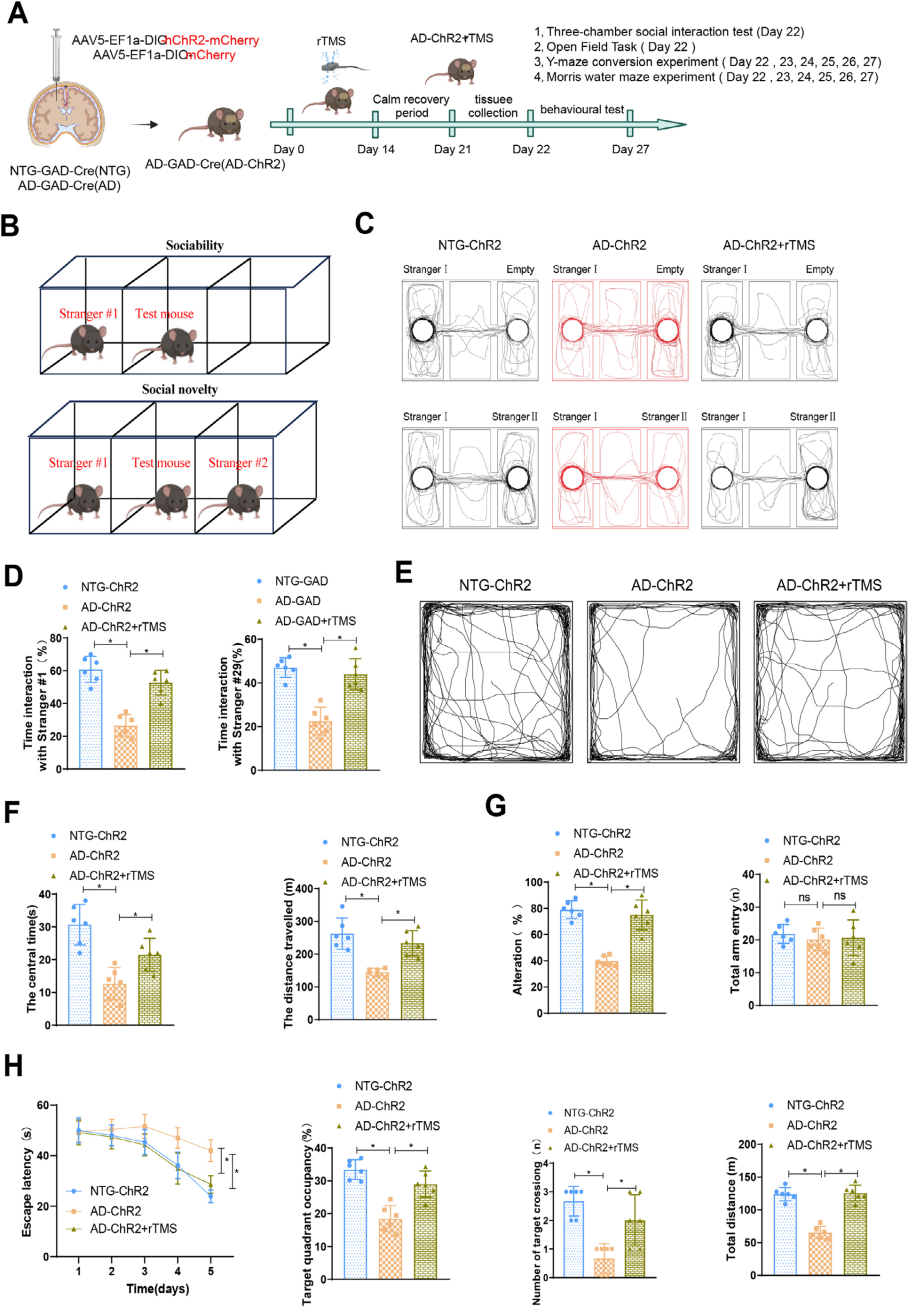
Influence of rTMS on behavioural changes of GABAergic neurons in 5xFAD mice. (A) Schematic illustrating the treatment process using rTMS on mice with 5xFAD as an AD model; (B) Schematic representation of the three‐chamber social behaviour test procedure; (C) Movement trajectories of mice in the three‐chamber social behaviour test chambers for both groups; (D) Bar chart summarising the time spent by mice in each chamber during the three‐chamber social behaviour test; (E) Movement trajectories of mice in the open field experiment; (F) Bar graphs summarising the time spent in the center of the arena, total distance travelled by mice in the open field experiment; (G) Bar graphs depicting the percentage of alternation and total arm entries in the Y‐maze test for all groups; (H) Bar graphs showing escape latency, target quadrant occupancy, target crossings and total distance travelled in the Morris water maze for all groups. *n* = 6, *p < 0.05.

Our previous research has demonstrated that rTMS can improve the impaired functionality of GABAergic neurons and downregulation of GABA expression in 5xFAD mice. Excessive Aβ levels can lead to neuronal dysfunction, reduce the activity of GABAergic interneurons, and ultimately contribute to the development of AD. To further investigate the role of GABAergic interneurons in the context of AD, we established a mouse model (AD‐GAD) targeting the general population of GABAergic neurons in the presence of amyloidosis through glutamic acid decarboxylase or GAD expression. By crossing heterozygous AD/NTG mice with homozygous GAD‐Cre animals, we generated AD‐GAD‐Cre mice, with NTG‐GAD‐Cre mice serving as controls. To explore the impact of rTMS on GABAergic neurons, we utilised Cre/Lox recombination technology to selectively express channelrhodopsin‐2 (ChR2) in the prefrontal cortex GABAergic interneurons of AD‐GAD‐Cre (AD) and NTG‐GAD‐Cre (NTG) mice (AD‐ChR2 and NTG‐ChR2). Subsequently, in vivo studies were conducted on AD‐ChR2 mice treated with rTMS, as outlined in Figure 3A.

To assess changes in social behaviour among the mouse groups, we performed behavioural testing. The order of behavioural tests was the three‐chamber social interaction test, open field test, Y‐maze alternation test and Morris water maze test. Each experimental group consisted of six mice. The three‐chamber social interaction test and open field test were performed on Day 22, followed by the Y‐maze alternation test and Morris water maze test, which were performed on alternate days after allowing the mice to recover. We evaluated their sociability and social novelty using the three‐chamber social behaviour test (Figure 3B). The results indicated that compared to the NTG‐ChR2 group, the AD‐ChR2 group showed significantly reduced interaction time with unfamiliar mouse #1 and unfamiliar mouse #2, which was partially ameliorated with rTMS therapy as evidenced by a significant increase in interaction time in the AD‐ChR2 + rTMS group (Figure 3C,D).

Subsequently, to evaluate anxiety and exploratory behaviour in mice, we conducted the open field test. The results revealed that the AD‐ChR2 group mice exhibited significantly lower time spent in the center of the arena and total distance travelled compared to the NTG‐ChR2 group. However, after rTMS treatment, the AD‐ChR2 group mice showed an increase in center time and distance travelled (Figure 3E,F).

In assessing spatial memory function in mice, we conducted the Y‐maze test and Morris water maze experiment. The results indicated a significant decrease in the percentage of alternation in the Y‐maze test for the AD‐ChR2 group compared to the AD‐ChR2 + rTMS group, while the AD‐ChR2 + rTMS group showed a partial recovery (Figure 3G). In the Morris water maze experiment, the AD‐ChR2 group exhibited prolonged escape latency, decreased occupancy of the target quadrant, reduced number of target crossings, and total distance travelled, whereas the AD‐ChR2 + rTMS group, post rTMS treatment, displayed reduced escape latency, increased target quadrant occupancy, enhanced number of target crossings, and total distance travelled, all significantly different from the AD group (Figure 3H). Additionally, without rTMS treatment, there were no significant differences in neural function among AD mice expressing ChR2 (Figure S4).

Furthermore, we evaluated the expression of pro‐inflammatory cytokines in the frontal cortex proteins of mice in each group using Western Blot analysis. The results revealed a significant upregulation of TNF‐α, IL‐1β and IL‐6 expression in the frontal cortex of AD‐ChR2 mice compared with NTG‐ChR2 mice. However, following rTMS treatment, the expression of these pro‐inflammatory cytokines was significantly suppressed (Figure S5A). RT‐qPCR results were consistent with Western Blot analysis, demonstrating the mRNA levels of the aforementioned factors (Figure S5B). In the absence of rTMS treatment, there were no significant differences in the inflammatory response among AD mice expressing ChR2 (Figure S5C,D).

In conclusion, rTMS can alleviate symptoms in 5xFAD mice targeting AD‐ChR2.

### 3.4 rTMS Stimulation of GABAergic Neurons Reduces Amyloid Plaque Deposition in AD Mice

**FIGURE 4:**
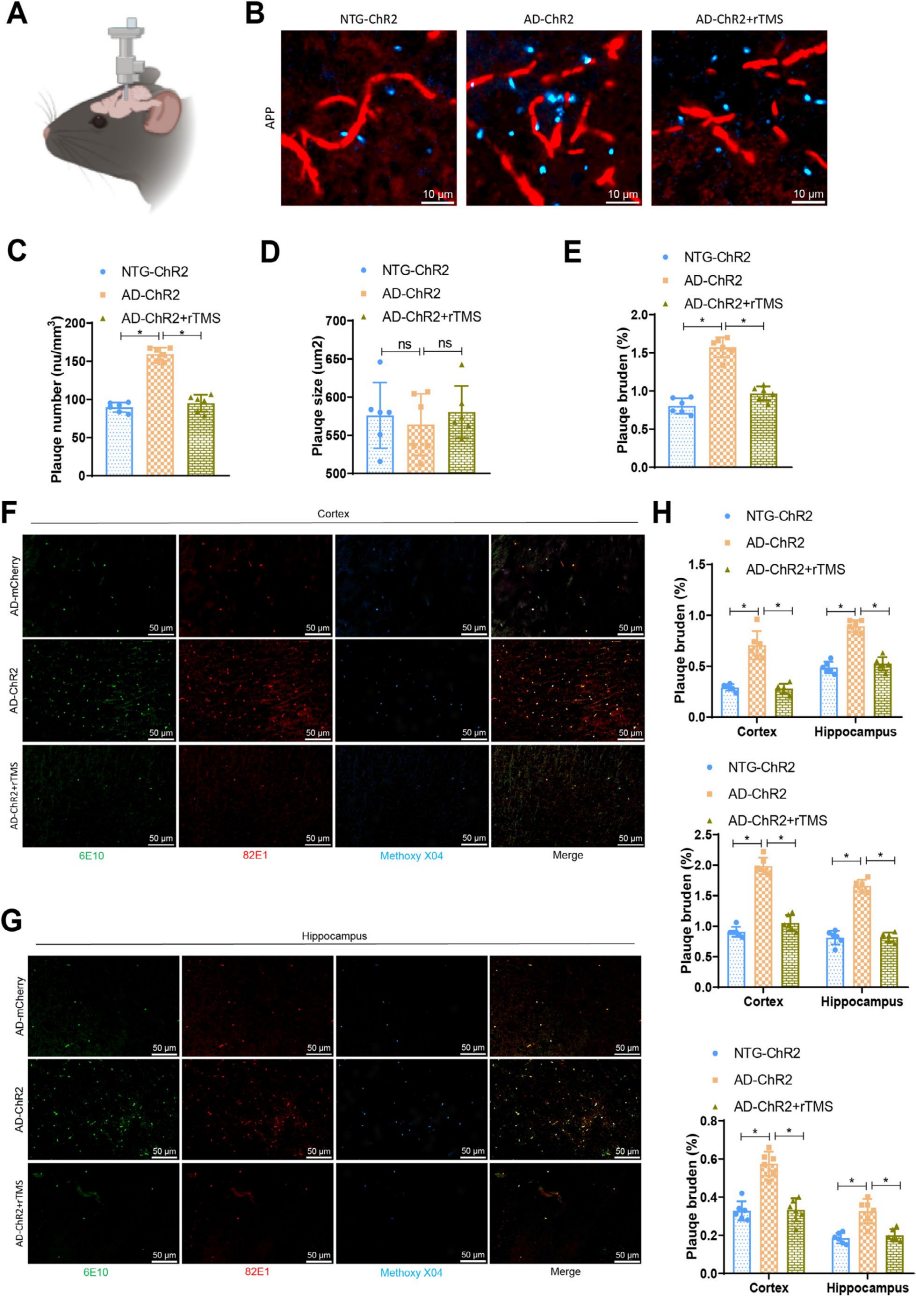
Influence of rTMS stimulation on GABAergic neurons in AD mice regarding the deposition of amyloid plaques. (A) Schematic illustration of mice undergoing multiphoton imaging; (B) typical multiphoton image of Methoxy‐XO4‐positive amyloid plaques (green) and blood vessels (red), with a scale bar of 10 μm; (C) number of amyloid plaques under different conditions; (D) size of amyloid plaques under different conditions; (E) burden of amyloid plaques under different conditions; (F, G) typical images of 6E10 (green), 82E1 (red) and Methoxy‐XO4 (blue) positive amyloid plaques in the brain tissue cortex (F) and hippocampus (G), with a scale bar of 50 μm; (H) load of 6E10, 82E1 and Methoxy‐XO4 positive amyloid plaques. *n* = 6, *p < 0.05.

After mounting a cranial window on the right posterior cortex, we further utilised Methoxy‐XO4 to label amyloid plaques and monitored them using multiphoton microscopy (Figure 4A,B). The AD‐ChR2 group exhibited a significant increase in amyloid plaque count compared to the NTG‐ChR2 group. Conversely, upon comparing with the AD‐ChR2 group, a noticeable reduction in amyloid plaque count was observed in the AD‐ChR2 + rTMS group following rTMS stimulation (Figure 4C). The plaque sizes were comparable among the three groups (Figure 4D). Moreover, in rTMS‐treated AD mice, both the quantity and size of amyloid plaques were significantly diminished (Figure 4E). These data suggest that rTMS stimulation of GABAergic neurons leads to a reduction in amyloid plaque deposition in AD mice. As multiphoton microscopy cannot access deeper brain regions like the hippocampus, we validated the amyloid plaque data in the larger cortical region and the hippocampus. Immunostaining was performed on postmortem brain tissues of the same AD mice using anti‐β‐amyloid antibodies 6E10, which recognise the N‐terminal of Aβ but not full‐length APP, and 82E1 for immunostaining, alongside Methoxy‐XO4 immunostaining. Methoxy‐XO4‐labelled amyloid plaques exhibited dense cores, with 6E10 and 82E1 decorating the periphery and core. rTMS stimulation significantly reduced amyloid plaque burden in the cortex and hippocampus. Immunoreactivity of 6E10 and 82E1 demonstrated that, compared to the AD‐ChR2 group, the AD‐ChR2 + rTMS group exhibited a substantial decrease in amyloid plaque burden under rTMS stimulation (Figure 4F,G). Methoxy‐XO4 data similarly indicated a marked reduction in plaque burden following rTMS stimulation (Figure 4H), consistent with the in vivo multiphoton microscopy results. Furthermore, AD mice expressing ChR2 without rTMS stimulation did not show a decrease in plaque deposition (Figure S6A–C).

In conclusion, rTMS stimulation of GABAergic neurons can ameliorate amyloid plaque deposition in AD mice.

### 3.5 Exploring the Mechanisms of rTMS in the Treatment of AD: Activation of Microglia and Clearance of Aβ

**FIGURE 5:**
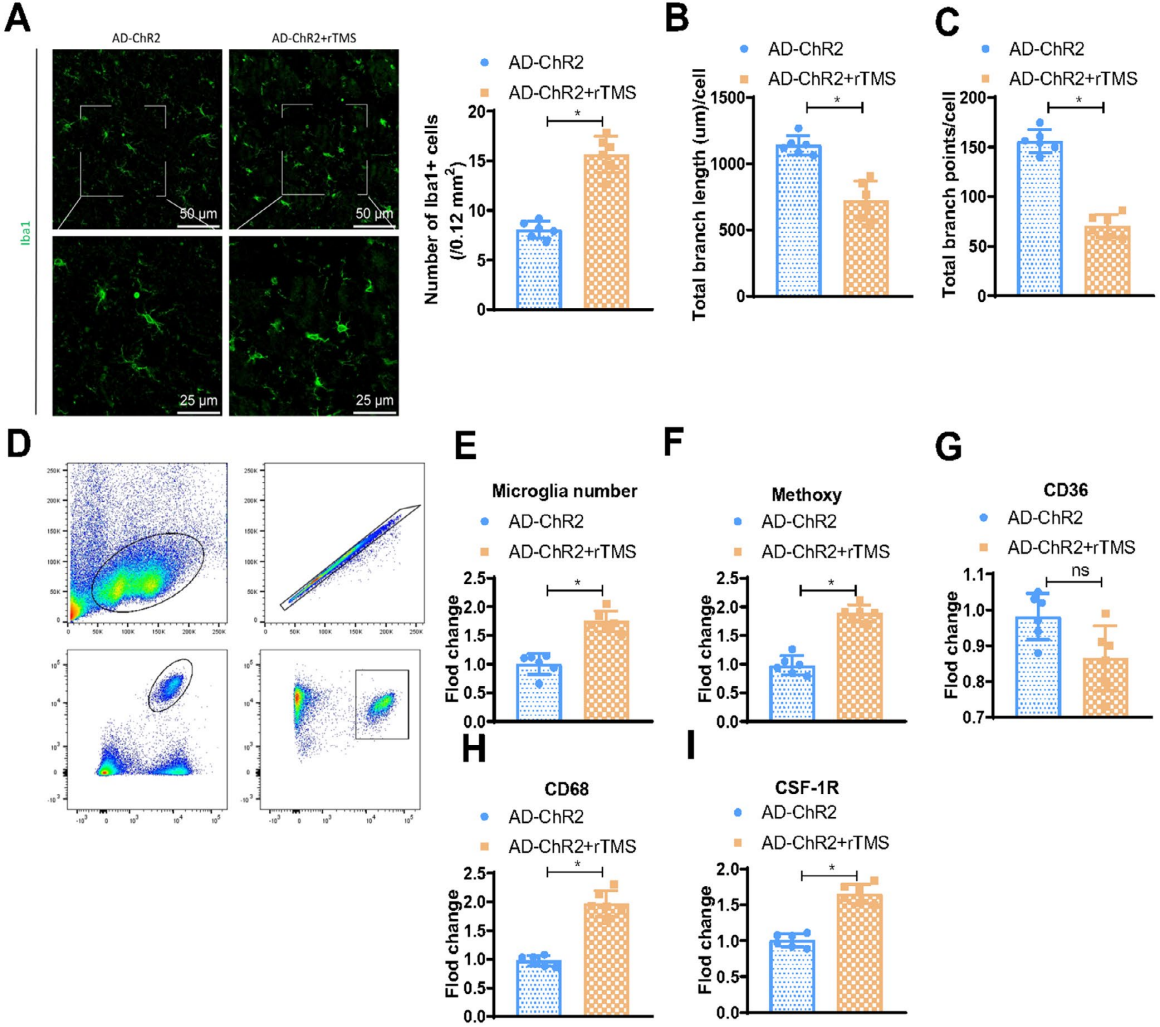
Impact of rTMS stimulation on the functionality of microglia in AD mice. (A) Three‐dimensional reconstruction images of Iba1 immunostaining and microglial cells, along with quantification of the number of Iba1‐positive cells. Scale bar is 40 μm (overview) and 20 μm (insets and renderings); (B, C) Semi‐automated quantitative analysis of cell morphology using IMARIS, including total process length of Iba1‐positive microglial cells (B) and number of branching points (C); (D) Demonstration of gating strategy for identifying CD11b^+^CD45^low^ microglia; (E, F) Percentage of Aβ‐engulfing microglia (Methoxy^−^XO4^+^ microglia), with quantification of microglial clusters (E) and Methoxy‐XO4^+^CD11b^+^CD45^low^ microglia (F); (G–I) Expression levels of microglia CD36 (G), CD68 (H) and CSF‐1R (I) quantified. *n* = 6, *p < 0.05.

Previous studies have indicated that in mice, microglia act as key regulators of neuronal activity and related behavioural responses [61, 63]. Our prior research has shown that rTMS stimulation can enhance the interaction between microglia and neurons. To further investigate the activity between GABAergic neurons and microglia in the treatment process of AD, we initially detected the activation of microglia by immunofluorescence staining of the microglial specific marker Ionised calcium‐binding adapter molecule 1 (Iba1). In the AD‐ChR2 + rTMS mouse model, the number of Iba1^+^ microglia was significantly higher after rTMS stimulation compared to the corresponding AD‐ChR2 mice (Figure 5A). As the morphology of microglia is linked to their activation state [64], we used semi‐automated quantitative morphological measurements in three‐dimensional (3D) imaging to analyse the morphology of microglia. The results revealed a significant reduction in processes (Figure 5B) and branching points (Figure 5C) in AD‐ChR2 mice after rTMS stimulation compared to AD‐ChR2 mice. To validate our observations on microglial density changes, we stained for Ki67 (a cell proliferation marker) and found a significant increase in Ki67 expression in hippocampal CA1 microglia after rTMS stimulation compared to the AD‐ChR2 group (Figure S7A).

In order to further assess the functionality of microglia, flow cytometry analysis was performed on cells isolated from fresh tissue, identifying microglia with CD11b and CD45 markers (Figure 5D). It was observed that rTMS treatment led to an increase in microglial numbers in AD‐ChR2 mice (Figure 5E), consistent with the immunofluorescence data. To investigate the phagocytic ability of microglia, we measured the percentage of methoxy‐XO4^+^CD11b^+^CD45^low^ viable microglia as well as the expression of the phagocytic markers CD36 and CD68. Compared to the AD‐ChR2 group, rTMS treatment resulted in an increased percentage of Methoxy‐XO4^+^CD11b^+^CD45^low^ microglia (Figure 5F) and higher expression of CD68 (Figure 5H), with no significant change in CD36 expression (Figure 5G). This suggests that the ability of microglia in the AD‐ChR2 + rTMS group to clear Aβ is enhanced. Furthermore, rTMS treatment upregulated the expression of the proliferation marker CSF‐1R in microglia (Figure 5I), consistent with the observed increase in microglial numbers. Additionally, AD mice expressing ChR2 without rTMS stimulation showed no significant differences in microglial numbers and phagocytic capacity (Figure S7B–E).

To investigate the relationship between microglial activation and AD treatment with rTMS, we intracranially injected minocycline, a tetracycline derivative widely used to inhibit microglial activity [65]. Results from the mouse model pre‐injected with minocycline showed a significant reduction in social interaction time with unfamiliar mice #1 and #2 in the AD‐rTMS+Mino group compared to the AD‐rTMS+Saline group (Figure S8A,B). Central time and total distance travelled in the arena were markedly decreased (Figure S8C,D). The percentage of alternation in the Y‐maze test was significantly reduced (Figure S8E). Escape latency increased while target quadrant occupancy and target crossings significantly decreased (Figure S8F).

In summary, these findings suggest that rTMS stimulation targeting GABAergic neurons enhances the proliferation of reactive microglia, induces a clustering phenotype around plaques, and facilitates microglial phagocytosis of Aβ, leading to Aβ clearance.

### 3.6 Validation and Expression Regulation of the Mechanism of Action of Cx3cl1 in GABAergic Neurons as Potential rTMS Treatment Targets for AD

**FIGURE 6:**
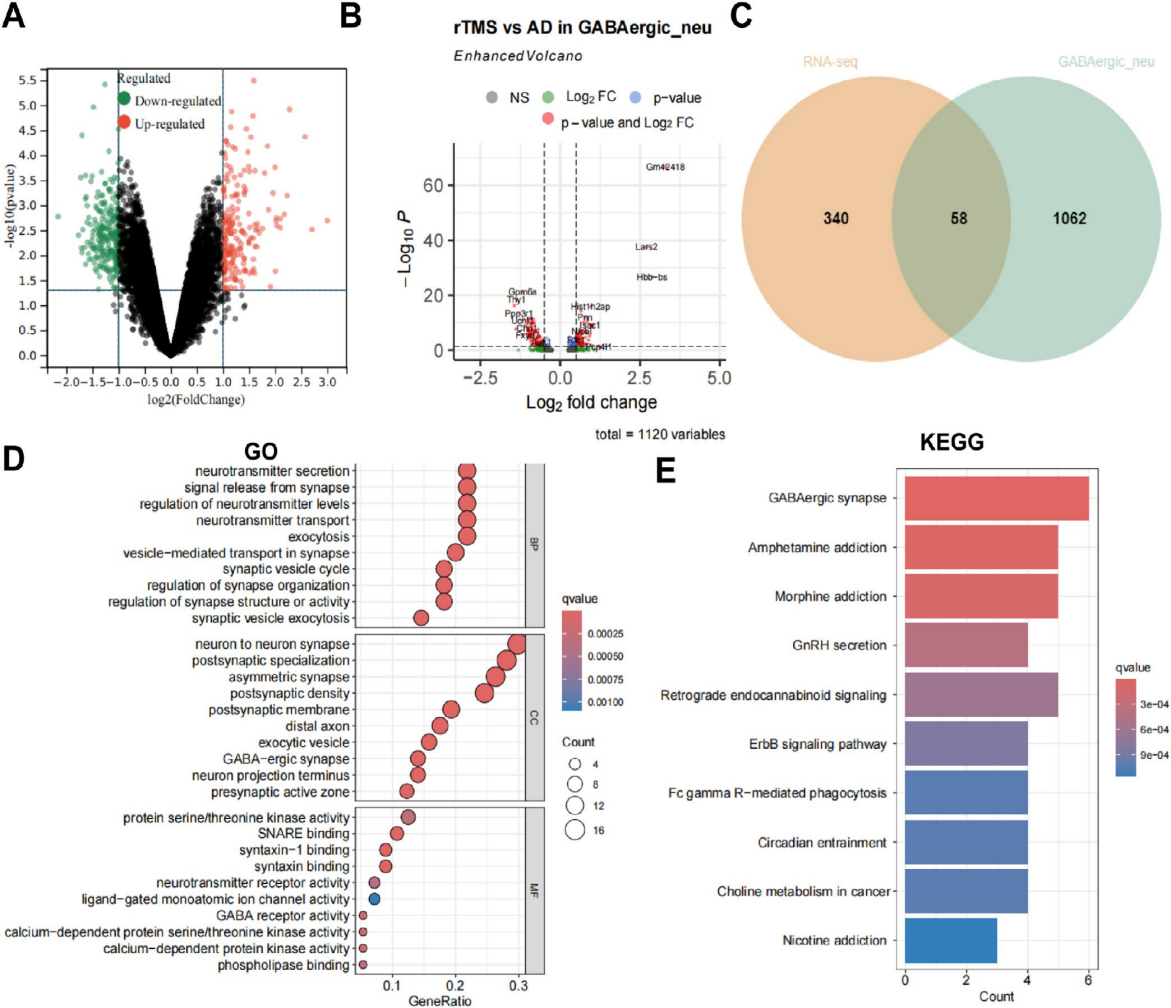
Cx3cl1 is a key gene in rTMS treatment of AD. (A) Volcano plot analysis of differentially expressed genes from transcriptome sequencing of cortical samples from 5xFAD mice treated with or without rTMS; (B) Differentially expressed genes in the GABAergic neuron subpopulation based on single‐cell transcriptome sequencing; (C) Venn diagram showing the intersection of the differentially expressed genes; (D) Bubble plot of GO enrichment results for 58 differentially expressed genes; (E) Histogram of KEGG enrichment results for 58 differentially expressed genes.

To further explore the mechanism of action of rTMS targeting GABAergic neurons for AD treatment, we conducted transcriptome‐sequencing analysis on cortical tissues of 5xFAD mice targeted with or without rTMS treatment. We identified 398 differentially expressed genes, comprising 185 upregulated and 213 downregulated genes (Figure 6A). Subsequently, by integrating differential marker genes of the AD and rTMS GABAergic neuron Neu1 subgroups from single‐cell transcriptome sequencing (Table S2), a total of 1120 differentially expressed genes were obtained (Figure 6B). By intersecting these gene sets, we identified 58 candidate genes (Figure 6C).

**FIGURE 7:**
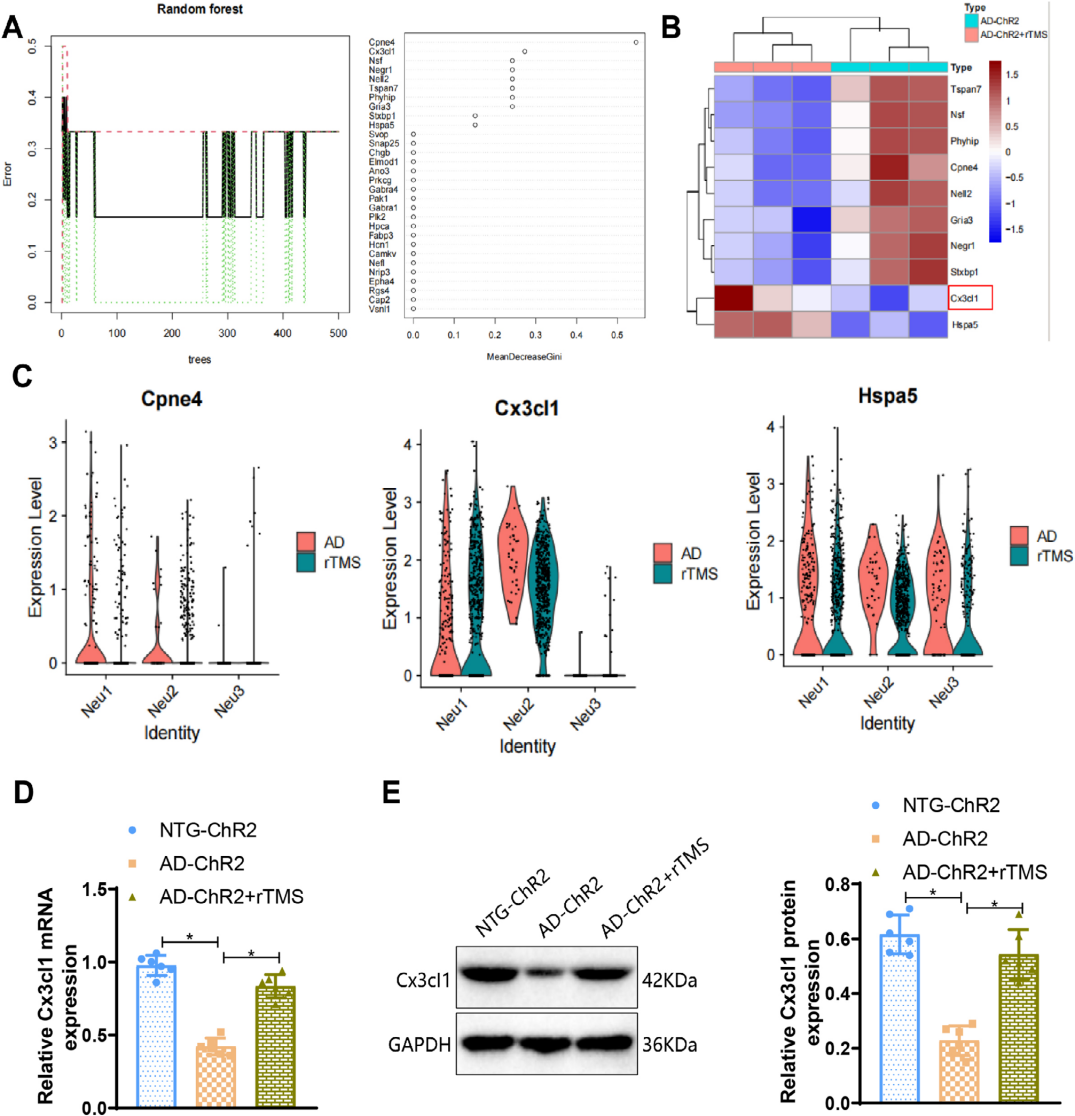
Cx3cl1 is a key gene in rTMS therapy for AD. (A) Analysis results of random forest algorithm applied to these 58 candidate genes; (B) transcriptome sequencing of frontal cortex samples from 5xFAD mice receiving or not receiving rTMS treatment, presenting expression heatmap of 10 candidate genes; (C) examination of the cell enrichment of Cpne4, Cx3cl1 and Hspa5 in neuronal cell subtypes; (D) detection of Cx3cl1 mRNA levels in the cortex of mice groups using RT‐qPCR; (E) assessment of Cx3cl1 protein levels in the cortex of mouse groups using Western Blot, accompanied by statistical bar graph presentation. *n* = 6, *p < 0.05.

We conducted a bioinformatics analysis of the above results and performed GO and KEGG pathway enrichment analysis on 58 candidate genes. Both the GO and KEGG results showed that GABA‐related pathways were involved (Figure 6D,E). To identify key targets for rTMS treatment of AD, we used the gene machine‐learning algorithm, Random Forest, to compute scores for these 58 candidate genes. Ten genes were scored, with Cpne4 and Cx3cl1 ranking higher (Figure 7A). Additionally, among these 10 genes, Cx3cl1 and Hspa5 showed high expression in the transcriptome sequencing results, while Cpne4 showed low expression (Figure 7B). Based on these three genes, we performed single‐cell transcriptome sequencing validation and found that only Cx3cl1 was highly expressed in the GABAergic neuron (Neu1) subpopulation after rTMS treatment (Figure 7C).

HSPA5 is a 70 kDa heat shock protein with a typical heat shock protein structure. It includes an N‐terminal ATPase domain, an intermediate ATP binding domain, and a C‐terminal protein‐binding domain. These domains allow HSPA5 to interact with other proteins and participate in protein folding and unfolding processes. Previous studies have confirmed that in many cell and animal models of Parkinson's disease, the expression of HSPA5 and other UPR proteins is elevated [66]. Consequently, we selected Cx3cl1 for further investigation. Additionally, existing literature has reported an increase in Cx3cl1 secretion in GABA neurons, leading to microglia activation [41].

To validate the bioinformatics analysis results, we utilised 5xFAD transgenic mice as an AD model and subjected them to rTMS therapy to observe the expression of Cx3cl1. RT‐qPCR and Western Blot analyses were conducted on samples from the frontal cortex of the mouse brains in each group. The results indicated a significant downregulation of Cx3cl1 expression in the AD‐ChR2 group compared to the NTG‐ChR2 group, whereas the AD‐ChR2 + rTMS group showed a restoration in Cx3cl1 expression levels following rTMS treatment (Figure 7D,E). Through bioinformatics analysis and molecular biology investigations in mice, we conclude that Cx3cl1 is a potential target for rTMS therapy in AD.

### 3.7 Morphological and Functional Changes of Microglia in rTMS Therapy: The Role of Cx3cl1‐Cx3cr1 Signalling Regulation

**FIGURE 8:**
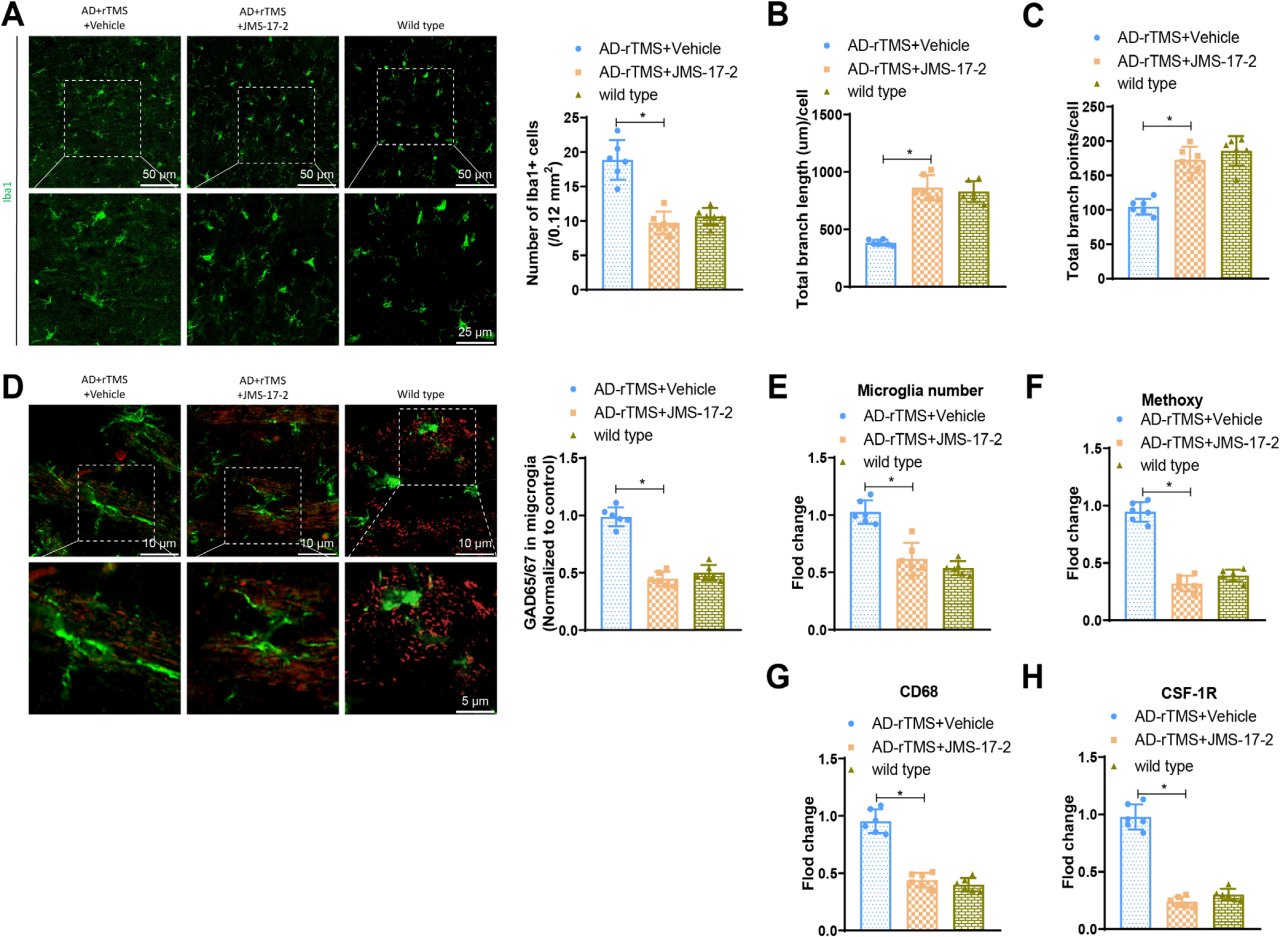
The impact of rTMS stimulation on the function of microglia in AD mice via the Cx3cl1‐Cx3cr1 signalling axis. (A) Illustrates representative images of Iba1 immunostaining and three‐dimensional reconstruction of microglial cells, along with quantification of the number of Iba1‐positive cells. Scale bar is 40 μm (overview) and 20 μm (insets and renderings); (B, C) semi‐automated morphological quantification analysis using IMARIS, including total process length and branch point numbers of Iba1‐positive microglial cells; (D) presents representative images of Iba1^+^ microglia (green) containing GAD65/67^+^ spots (red) and their quantitative results (10 cells per group, 5 mice per group). Scale bar is 10 μm (overview) and 5 μm (insets and renderings); (E, F) quantification of Aβ‐phagocytic microglia (Methoxy‐XO4^+^ microglia) ratio, as well as quantification of microglial clusters (E) and Methoxy‐XO4^+^CD11b^+^CD45^low^ microglia (F); (G, H) quantification of CD68 (G) and CSF‐1R (H) expression in microglia. *n* = 6, *p < 0.05.

It is widely recognised that Cx3cl1/fractalkine is a secreted chemokine factor specifically expressed in neurons, which activates microglia by binding to its receptor Cx3cr1 [67]. Therefore, we further investigated the interaction of Cx3cl1‐Cx3cr1 signalling between neurons and microglia. In mice, we injected the Cx3cr1‐specific antagonist JMS‐17‐2 or a control, followed by high‐frequency rTMS treatment (AD‐ChR2 + rTMS + Vehicle group, AD‐ChR2 + rTMS + JMS‐17‐2). The results showed a significant decrease in the number of Iba1^+^ microglia in the AD‐ChR2 + rTMS model mice treated with JMS‐17‐2 compared with the respective control group mice (Figure 8A). Additionally, the number of Iba1+ microglia in the AD‐ChR2 + rTMS + JMS‐17‐2 group was not significantly different from that in healthy wild‐type mice. The total length of molecular points was significantly increased (Figure 8B), and the number of branching points also significantly increased (Figure 8C), with no significant difference from healthy wild‐type mice levels. Furthermore, immunofluorescence staining further revealed that the phagocytic rate of GAD65/67 microglia in mice pretreated with the Cx3cr1 antagonist was significantly lower than in the control group (Figure 8D).

To further assess the function of microglia using flow cytometry, consistent with in vivo results, JMS‐17‐2 treatment resulted in a decrease in the number of microglia in AD‐ChR2 mice (Figure 8E). Additionally, JMS‐17‐2 treatment also led to a weakening of the phagocytic ability of microglia, as evidenced by a decrease in the percentage of Methoxy‐XO4^+^CD11b^+^CD45^low^ microglia (Figure 8F), as well as reduced expression of CD68 and CSF‐1R (Figure 8G,H). In conclusion, these results indicate that rTMS therapy induces changes in the morphological features and phagocytic ability of microglia through the Cx3cl1‐Cx3cr1 signalling axis.

### 3.8 rTMS Stimulation Reduces Amyloid Plaque Deposition in AD Mice by Modulating GABAergic Neurons

**FIGURE 9:**
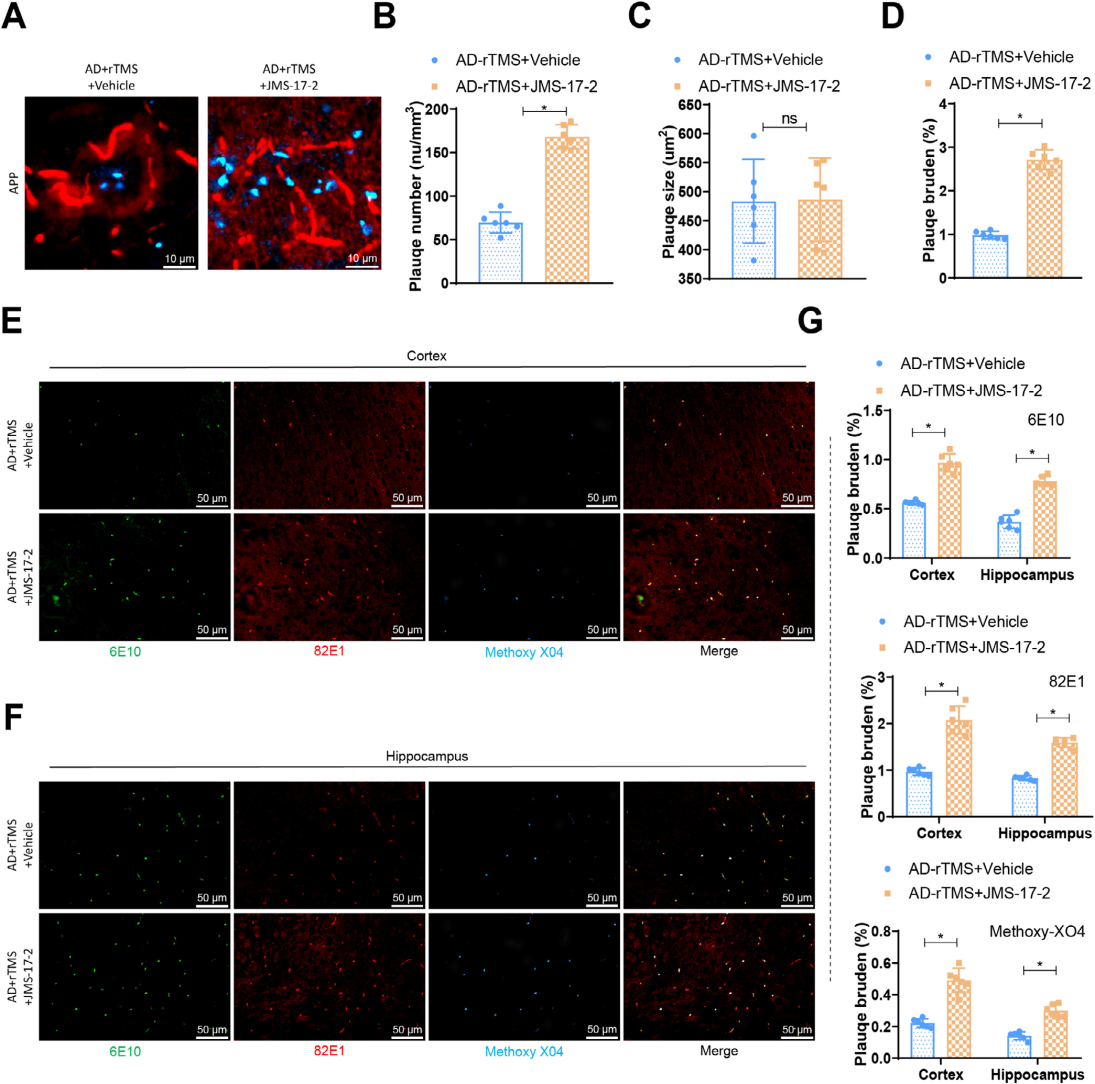
The impact of rTMS stimulation on amyloid plaque deposition in a Cx3cl1‐Cx3cr1 axis‐dependent manner. (A) Representative multiphoton images displaying Methoxy‐XO4‐positive amyloid plaques (cyan) and blood vessels (red); (B) quantity of amyloid plaques under different conditions; (C) size of amyloid plaques under similar conditions; (D) burden of amyloid plaques under different scenarios; (E, F) representative images of 6E10 (green), 82E1 (red) and Methoxy‐XO4 (blue) positive amyloid plaques in the cortical (E) and hippocampal (F) brain tissues, scale bar = 50 μm; (G) load of 6E10, 82E1 and Methoxy‐XO4 positive amyloid plaques. *n* = 6, *p < 0.05.

In this study, we investigated the impact of rTMS stimulation on GABAergic neurons on amyloid plaque deposition in AD mice. A significant increase in plaque quantity was observed in the AD‐rTMS + JMS‐17‐2 group compared to the AD‐rTMS + Vehicle group (Figure 9A,B), while plaque size remained consistent (Figure 9C). The amyloid plaque burden in terms of quantity and size was notably elevated in the AD mice of the AD‐rTMS + JMS‐17‐2 group (Figure 9D). Immunostaining of postmortem brain tissues from the same AD mice revealed a marked increase in immune reactivity of 6E10 and 82E1 in the AD‐rTMS + JMS‐17‐2 group, indicating a significant rise in amyloid plaque burden. Additionally, methoxy‐XO4 data demonstrated an increase in plaque load following rTMS stimulation (Figure 9E–G).

Further behavioural observations of the mice were conducted. Results indicated that the AD‐rTMS + JMS‐17‐2 group displayed a significant decrease in the time spent with unfamiliar mice #1 and #2 compared with the AD‐rTMS + Vehicle group (Figure S9A,B). Moreover, reduced time spent in the center and total distance travelled in the arena were observed (Figure S9C,D). The Y‐maze test revealed a significant decrease in alternation percentage (Figure S9E), prolonged escape latency and a notable reduction in target quadrant occupancy and target crosses (Figure S9F).

These findings suggest that rTMS treatment contributes to improving 5xFAD mice, and its efficacy depends on the Cx3cl1‐Cx3cr1 signalling axis.

## 4 Discussion

An in‐depth investigation into the mechanisms of cognitive function recovery enhances the depth and breadth of research in the field of neuroscience [68]. This study confirmed the positive impact of rTMS therapy on cognitive function in an AD mouse model, highlighting the potential of this non‐invasive neuroregulation technique in the treatment of cognitive impairment. In comparison to previous human studies, our research, through detailed analysis of mouse brain tissue, provides a more specific and intricate mechanistic explanation, thus laying a more solid foundation for future clinical investigations.

Utilising scRNA‐seq technology, we revealed specific responses of brain cell types, particularly changes in the expression patterns of GABAergic neurons. In contrast to past studies, our research offers a more comprehensive quantitative description of the responses of these cell types, elucidating the mechanisms by which rTMS therapy regulates neuronal function [69, 70, 71]. This cellular‐level study contributes to a better understanding of the roles of different types of neurons in cognitive function recovery.

In this study, ligand‐receptor interaction analysis uncovered the impact of rTMS on intracranial signalling pathways, particularly through the Cx3cl1‐Cx3cr1 axis of signal transduction. Compared with prior research, our study highlights the investigation of cell–cell communication and signal transduction, revealing the mechanisms by which rTMS therapy regulates interactions among cells [9, 17, 72]. This comprehensive study of signal pathways provides a new profound understanding for unravelling the mechanisms underlying the improvement of cognitive function in AD, offering important insights for future therapeutic strategies.

Amyloid plaques hold significant importance in the pathophysiology of AD, and rTMS therapy promotes cognitive function improvement by reducing the number and size of amyloid plaques [73]. In comparison to existing relevant research, our study elaborates in greater detail how rTMS stimulates GABAergic neurons to enhance microglial clearance of Aβ, delving into the inherent mechanisms of reducing amyloid plaque deposition, thus providing new insights for AD treatment [74].

Neuroinflammation plays a crucial role in the development of AD, and this study observed that rTMS therapy reduces the expression levels of neuroinflammation markers. A distinguishing feature of our research is the emphasis on changes in the morphological characteristics and phagocytic function of microglia [31, 69, 75]. This indicates that rTMS therapy not only regulates cell function but also inhibits the progression of neuroinflammation by influencing cell morphology characteristics, offering a new perspective and value for a detailed understanding of the mechanisms behind AD treatment.

In summary, this study reveals the key role of rTMS in the restoration of cognitive function in AD through the activation of GABAergic neurons and mediation of the Cx3cl1‐Cx3cr1 axis. This finding not only enhances our understanding of the pathological mechanisms of AD but also presents new therapeutic targets and theoretical foundations for the non‐invasive treatment of AD. However, the study has certain limitations, such as differences between mouse models and human AD pathology, as well as the complexity of interpreting single‐cell data, which need further exploration in future research. Looking ahead, we anticipate transitioning the application of rTMS in AD treatment from the laboratory to clinical practice through broader experimental validation and in‐depth mechanistic studies, aiming to provide more effective treatment options for AD patients.

The demonstrated potential and mechanistic research of rTMS in AD treatment in this study hold significant scientific and clinical value. Firstly, treatment with rTMS in the 5xFAD mouse model successfully improved cognitive functions, including social ability, spatial memory and exploratory behaviour. This opens new avenues for the application of neuromodulation techniques in mental disorders and neuropathic pain. Secondly, the application of scRNA‐seq technology revealed transcriptomic changes in brain cells following rTMS treatment, particularly in the regulation of gene expression related to the GABAergic neuron‐mediated Cx3cl1‐Cx3cr1 axis. This provides a deeper understanding of intercellular signalling and communication, laying the groundwork for exploring new therapeutic targets within the brain.

Nevertheless, the study also presents some limitations. Firstly, the results using mouse models as research subjects may not be directly generalisable to human patients, requiring further translational medical research to validate the feasibility of clinical applications. Secondly, potential biases or unconsidered factors in the experimental design necessitate more control experiments and in‐depth analysis to validate the reliability of the results. Additionally, this study only used male mice, which limits the generalisability of the findings. Studies suggest that female mice may exhibit more severe pathological features in AD models, such as higher Aβ deposition and more significant cognitive decline. Using only male mice may not fully reflect gender differences in AD. Oestrogen may have neuroprotective effects, and hormonal fluctuations in female mice could affect AD pathology. The use of only male mice overlooks these hormone‐related effects [76]. Finally, we need to consider the potential comorbid depression that cannot be detected in animal models of dementia. In this case, the effects of non‐invasive neuroregulation techniques, including rTMS, might be insufficient [77], which requires further exploration and analysis. Furthermore, future research should focus on potential adverse reactions and associated complications that might arise in the translational application of cognitive rehabilitation in humans [78].

Future prospects include further exploring the mechanisms of rTMS in AD treatment, especially in terms of cellular‐level interactions and signal transduction pathways. Additionally, clinical trials could be considered to verify the efficacy and safety of rTMS in human patients and explore its application in personalised treatment. Ultimately, it is hoped that this study will offer new insights and methods for the development of neurodegenerative disease treatment and make more positive contributions to improving patients' quality of life.

## Author Contributions

Y.K. and J.L. contributed equally to this work. Y.K. and J.L. designed and performed experiments, analysed data and drafted the manuscript. Y.W., J.W. and J.W. assisted with data acquisition and animal experiments. C.Z. provided technical support and assisted with scRNA‐seq analysis. R.C. and T.Z. supervised the project, provided critical revisions and secured funding. All authors read and approved the final manuscript.

## Ethics Statement

All mouse experiments conducted in this study adhered to international and domestic ethical guidelines and regulations regarding experimental animals. The experimental procedures were approved by the Animal Ethics Committee of Hebei Medical University (Approval Number: IACUC‐Hebmu‐2,023,054). We ensured that all experimental animals were treated with respect and humane care throughout the research. The housing and handling of all mice were carried out in conditions aimed at minimising their pain and stress. Following the conclusion of the experiments, the mice were humanely handled. We express gratitude to Beijing Vital River Laboratory Animal Technology Co. Ltd. and Jackson Laboratory for providing the experimental animals for this study. Furthermore, we value and respect all individuals involved in this research, as well as each experimental animal, ensuring strict adherence to research ethics principles.

## Consent

The authors have nothing to report.

## Conflicts of Interest

The authors declare no conflicts of interest.

## Supporting information


**Figure S1.** Classification of variable genes and cell components in brain tissue samples of rTMS model using scRNA‐seq analysis. (A) Violin plots showing the number of genes (nFeature_RNA), mRNA molecules (nCount_RNA) and percentage of mitochondrial genes (percent.mt) for each cell in scRNA‐seq data; (B) Identification of highly variable genes among 31,053 genes using variance analysis (red dots represent high variability, black dots represent stable genes, showing the top 10 most variable genes); (C) Selection of the top 17 PCs using quantitative Elbow analysis; (D) t‐SNE clustering plot demonstrating the distribution of cell clusters after batch effect correction; (E) tSNE plot illustrating the expression of marker genes for major cell types detected in this study, with red indicating high expression and grey indicating low expression; (F) t‐SNE clustering plot displaying the distribution of 8 cell clusters from 5xFAD mice and rTMS model mice samples after batch effect correction.

**Figure S2.** Analysis of cell subpopulations and pseudotime analysis during the rTMS stimulation in the AD treatment process. (A) Subpopulation analysis of microglia, astrocytes and OPCs; (B) Trajectory order of cell subpopulations arranged by pseudotime values, with darker colours indicating lower pseudotime values (distance from the root node); (C) Cluster distribution of microglia, astrocytes and OPCs subpopulations on the developmental tree, with different colours representing different cell subpopulations.

**Figure S3.** Analysis of neuronal subpopulations and pseudotime. (A) Subpopulation analysis of neurons; (B) trajectory order of neuronal cell groups arranged by pseudotime values, with darker colours indicating lower pseudotime values (distance from the root node); (C) cluster distribution of neuronal subpopulations on the developmental tree, with different colours representing different neuronal subpopulations; (D) expression of representative genes for GABAergic neurons and glutamatergic neurons in neuronal subpopulations; (E) heatmap illustrating the expression levels of representative genes for GABAergic neurons and glutamatergic neurons, with colours ranging from blue to red indicating low to high relative expression levels.

**Figure S4.** Impact of isolated ChR2 expression on behavioural changes in 5xFAD mice. (A) Track plots of mouse behaviour in the three‐chamber social behaviour test; (B) bar graph of the duration spent by mice in each chamber in the three‐chamber social behaviour test; (C) track plots of mouse behaviour in the open field test; (D) bar graphs depicting the time spent in the center of the arena, total distance travelled by mice in the open field test; (E) bar graph showing the percentage of alternation and total arm entries in the Y‐maze test for each group; (F) bar graphs illustrating the escape latency, target quadrant occupancy, target crossings and overall movement distance in the Morris water maze test. *n* = 6, ns indicates *p* > 0.05.

**Figure S5.** Influence of rTMS treatment on neuroinflammation in 5xFAD mice. (A) RT‐qPCR analysis of TNF‐α, IL‐1β and IL‐6 mRNA levels in the frontal cortex of mice in each group; (B) Western blot analysis of TNF‐α, IL‐1β and IL‐6 protein levels in the frontal cortex of mice in each group; (C) RT‐qPCR examination of TNF‐α, IL‐1β and IL‐6 mRNA levels in the frontal cortex of mice in each group; (D) Western blot evaluation of TNF‐α, IL‐1β and IL‐6 protein levels in the frontal cortex of mice in each group. *n* = 6, *p < 0.05, ns indicates *p* > 0.05.

**Figure S6.** Impact of isolated ChR2 expression on plaque deposition in AD mouse models. (A–C) Typical images and statistical data of 6E10 (green), 82E1 (red) and Methoxy‐XO4 (blue) positive amyloid plaques in the cortex (A) and hippocampus (B), scale bar = 50 μm. *n* = 6, ns indicates *p* > 0.05.

**Figure S7.** Functional impact of rTMS stimulation on microglia in AD mice. (A) Representative images of Ki67 (red), Iba1 (green) and DAPI (blue) immunostaining, scale bar = 10 μm; (B, C) quantification of Aβ‐phagocytic microglia (Methoxy‐XO4^+^ microglia), quantitative analysis of the microglial population (B) and Methoxy‐XO4^+^CD11b^+^CD45^low^ microglial cells (C); (D, E) quantification of microglial CD68 (D) and CSF‐1R (E) expression levels. *n* = 6, *p < 0.05, ns indicates *p* > 0.05.

**Figure S8.** rTMS stimulation delays AD progression depending on activated microglia. (A) Track plots of mouse behaviour in the three‐chamber social behaviour test; (B) bar graph presenting the duration of stay in each chamber during the three‐chamber social behaviour test for different groups of mice; (C) track plots of mouse behaviour in the open field test; (D) bar graph showing the time spent in the center of the arena and total distance travelled by mice; (E) bar graph displaying the percentage of alternation and total arm entries in the Y‐maze test for different groups of mice; (F) bar graphs illustrating the escape latency, target quadrant occupancy, target crossings and total movement distance in the Morris water maze test. *n* = 6, *p < 0.05, ns indicates *p* > 0.05.

**Figure S9.** JMS‐17‐2 impact on rTMS stimulation delaying AD. (A) Track plots of mouse behaviour in the three‐chamber social behaviour test for different groups of mice; (B) bar graph of the duration of stay in each chamber during the three‐chamber social behaviour test; (C) track plots of mouse behaviour in the open field test; (D) bar graph showing the time spent in the center of the arena and total distance travelled by mice; (E) bar graph displaying the percentage of alternation and total arm entries in the Y‐maze test; (F) bar graphs illustrating the escape latency, target quadrant occupancy, target crossings and total movement distance in the Morris water maze test. *n* = 6, *p < 0.05, ns indicates *p* > 0.05.

**Table S1.** RT‐qPCR primer sequence.

**Table S2.** Differentially expressed genes in GABAergic neurons after rTMS treatment.

## Data Availability

The datasets generated and/or analysed during the current study are not publicly available due to privacy and confidentiality agreements with the participants but are available from the corresponding author on reasonable request.
