# Supplementary figures and images for: Repetitive Transcranial Magnetic Stimulation Induces Cognitive Recovery in Alzheimer's Disease via GABAergic Neuron Activation of the Cx3cl1‐Cx3cr1 Axis
Source: Cell Prolif. 2025 May 25;58(12):e70061. doi: 10.1111/cpr.70061 (PMC12686124; doi:10.1111/cpr.70061)

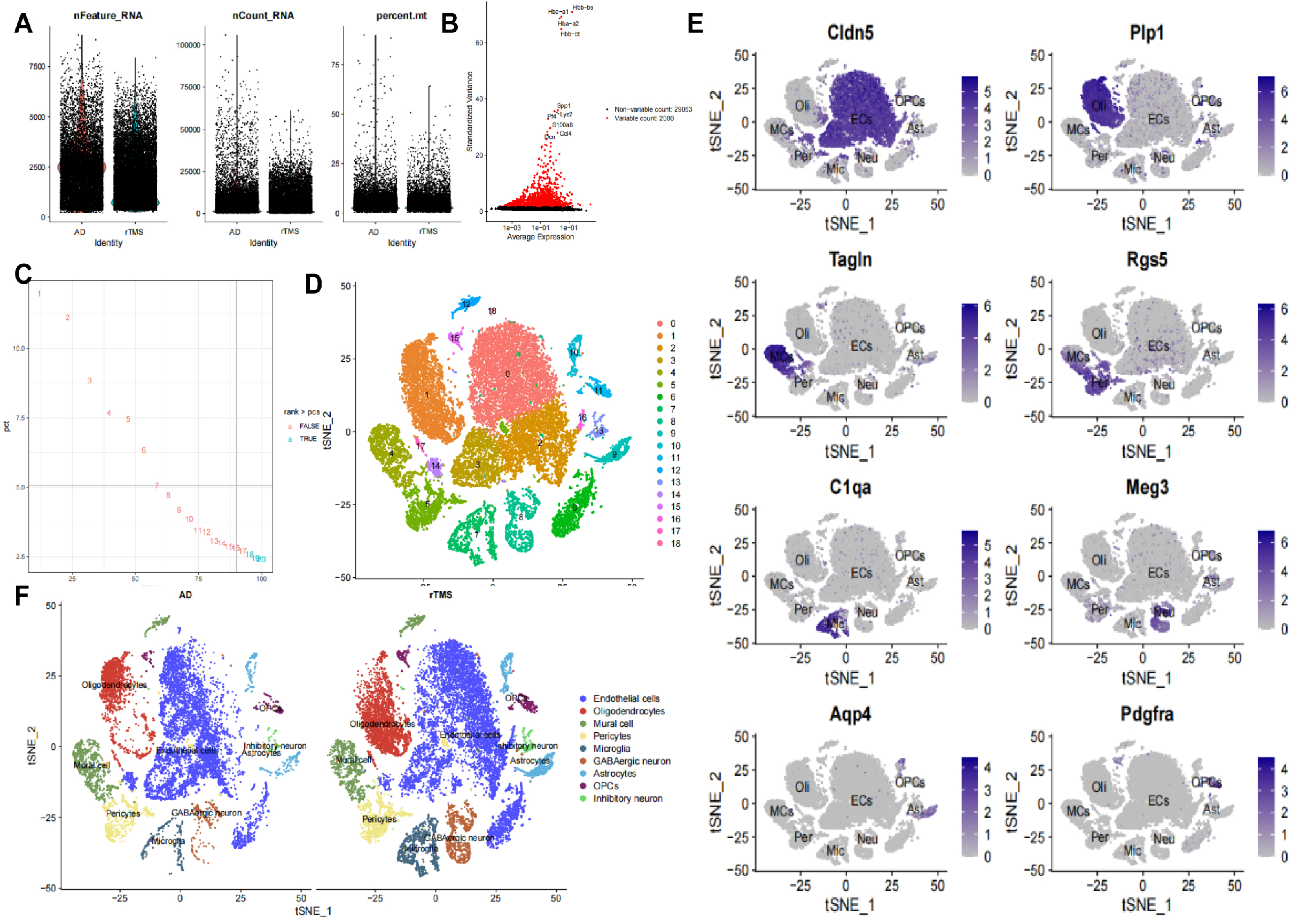

Supplement: Supplementary file 1 — Figure S1. Classification of variable genes and cell components in brain tissue samples of rTMS model using scRNA‐seq analysis. (A) Violin plots showing the number of genes (nFeature_RNA), mRNA molecules (nCount_RNA) and percentage of mitochondrial genes (percent.mt) for each cell in scRNA‐seq data; (B) Identification of highly variable genes among 31,053 genes using variance analysis (red dots represent high variability, black dots represent stable genes, showing the top 10 most variable genes); (C) Selection of the top 17 PCs using quantitative Elbow analysis; (D) t‐SNE clustering plot demonstrating the distribution of cell clusters after batch effect correction; (E) tSNE plot illustrating the expression of marker genes for major cell types detected in this study, with red indicating high expression and grey indicating low expression; (F) t‐SNE clustering plot displaying the distribution of 8 cell clusters from 5xFAD mice and rTMS model mice samples after batch effect correction. [file CPR-58-e70061-s002.jpg]

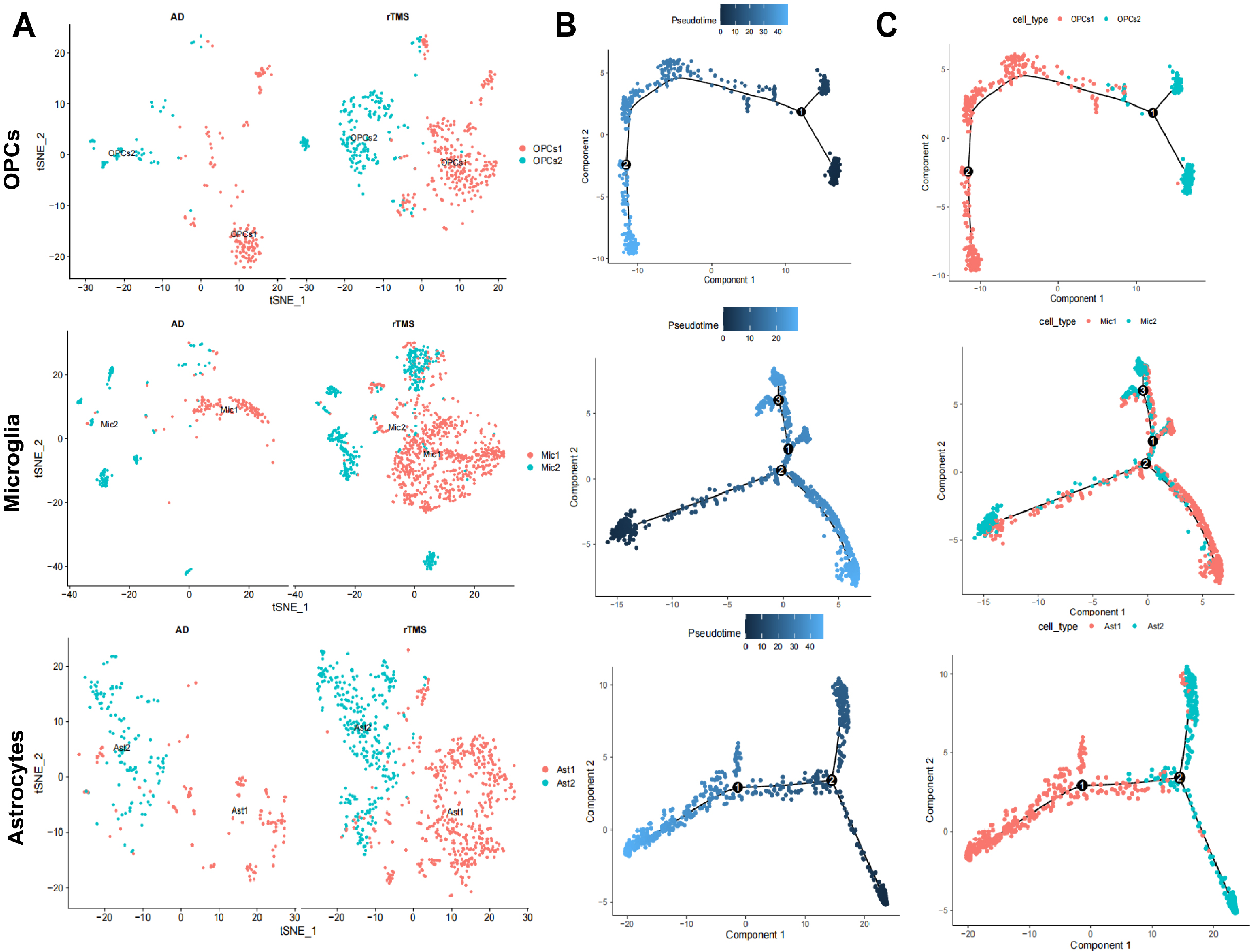

Supplement: Supplementary file 2 — Figure S2. Analysis of cell subpopulations and pseudotime analysis during the rTMS stimulation in the AD treatment process. (A) Subpopulation analysis of microglia, astrocytes and OPCs; (B) Trajectory order of cell subpopulations arranged by pseudotime values, with darker colours indicating lower pseudotime values (distance from the root node); (C) Cluster distribution of microglia, astrocytes and OPCs subpopulations on the developmental tree, with different colours representing different cell subpopulations. [file CPR-58-e70061-s001.jpg]

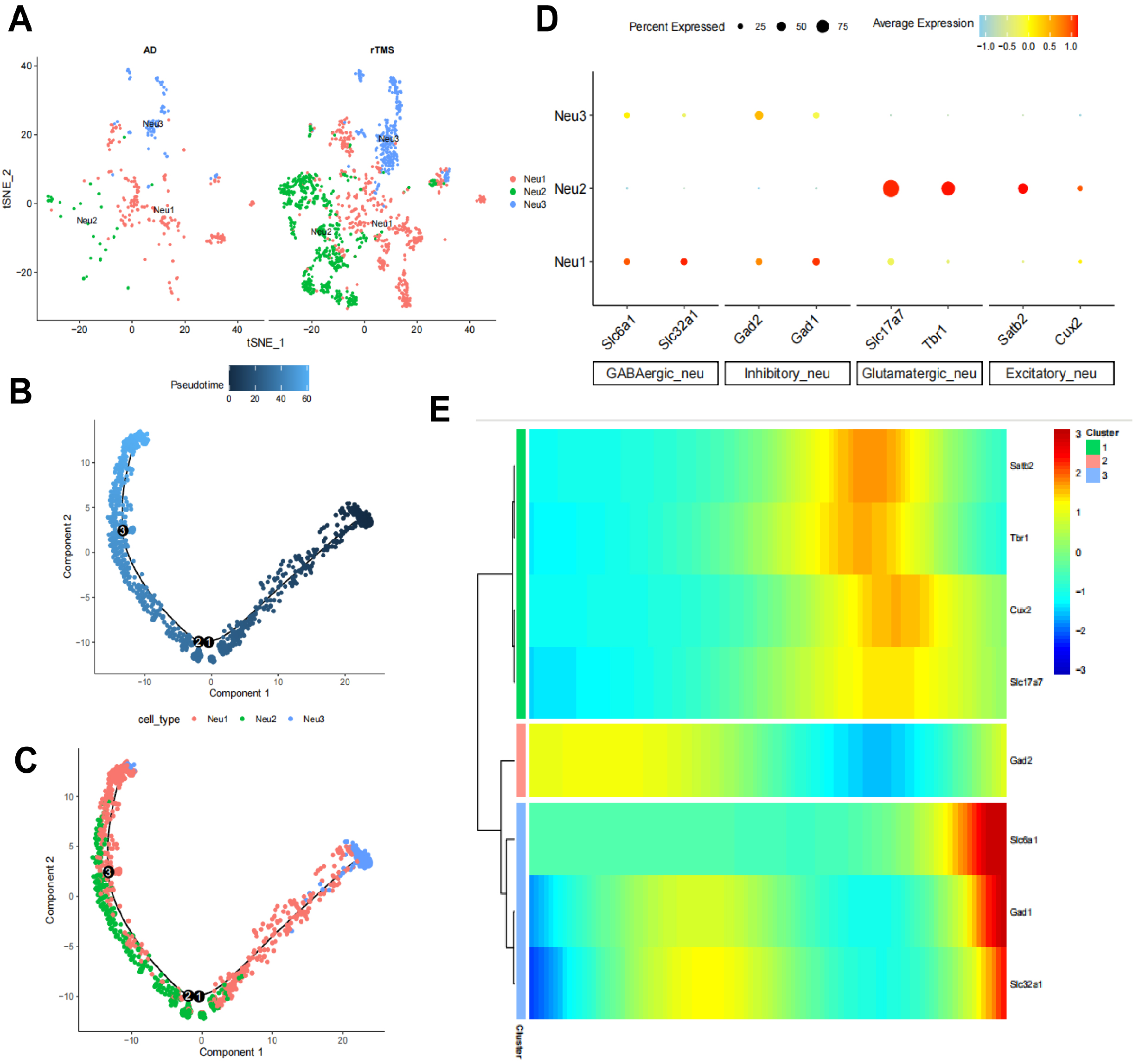

Supplement: Supplementary file 3 — Figure S3. Analysis of neuronal subpopulations and pseudotime. (A) Subpopulation analysis of neurons; (B) trajectory order of neuronal cell groups arranged by pseudotime values, with darker colours indicating lower pseudotime values (distance from the root node); (C) cluster distribution of neuronal subpopulations on the developmental tree, with different colours representing different neuronal subpopulations; (D) expression of representative genes for GABAergic neurons and glutamatergic neurons in neuronal subpopulations; (E) heatmap illustrating the expression levels of representative genes for GABAergic neurons and glutamatergic neurons, with colours ranging from blue to red indicating low to high relative expression levels. [file CPR-58-e70061-s005.jpg]

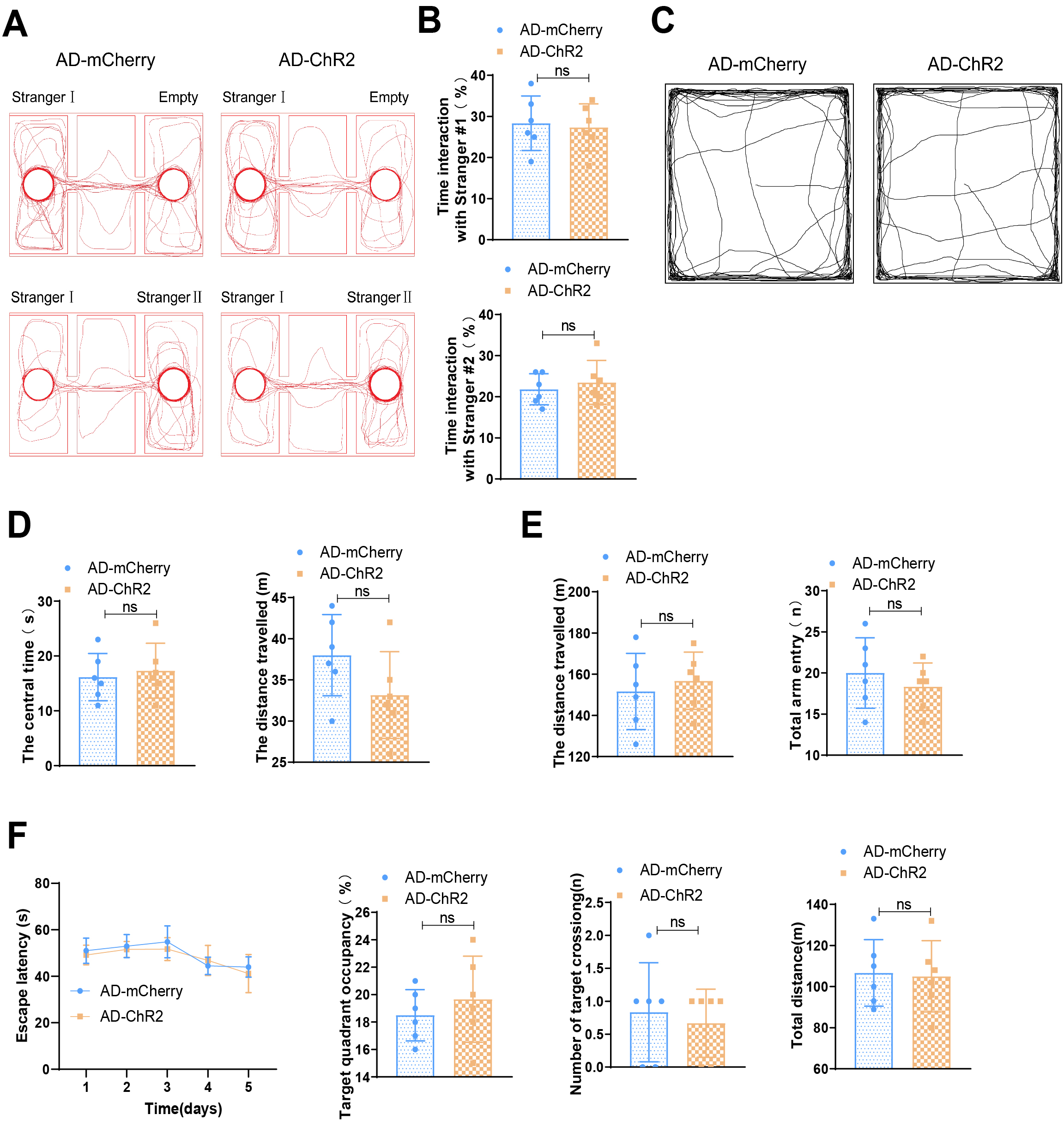

Supplement: Supplementary file 4 — Figure S4. Impact of isolated ChR2 expression on behavioural changes in 5xFAD mice. (A) Track plots of mouse behaviour in the three‐chamber social behaviour test; (B) bar graph of the duration spent by mice in each chamber in the three‐chamber social behaviour test; (C) track plots of mouse behaviour in the open field test; (D) bar graphs depicting the time spent in the center of the arena, total distance travelled by mice in the open field test; (E) bar graph showing the percentage of alternation and total arm entries in the Y‐maze test for each group; (F) bar graphs illustrating the escape latency, target quadrant occupancy, target crossings and overall movement distance in the Morris water maze test. n = 6, ns indicates p > 0.05. [file CPR-58-e70061-s010.jpg]

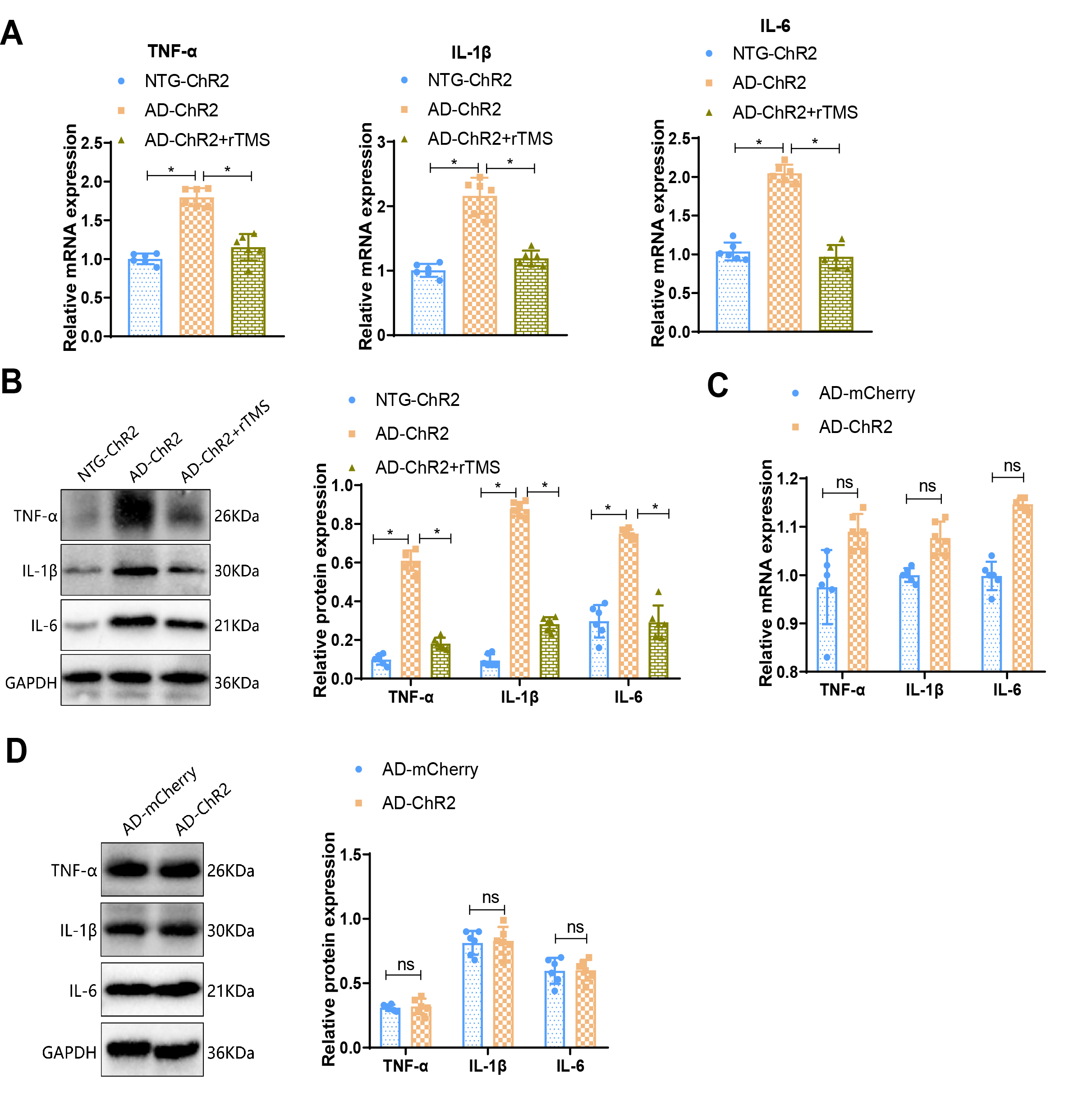

Supplement: Supplementary file 5 — Figure S5. Influence of rTMS treatment on neuroinflammation in 5xFAD mice. (A) RT‐qPCR analysis of TNF‐α, IL‐1β and IL‐6 mRNA levels in the frontal cortex of mice in each group; (B) Western blot analysis of TNF‐α, IL‐1β and IL‐6 protein levels in the frontal cortex of mice in each group; (C) RT‐qPCR examination of TNF‐α, IL‐1β and IL‐6 mRNA levels in the frontal cortex of mice in each group; (D) Western blot evaluation of TNF‐α, IL‐1β and IL‐6 protein levels in the frontal cortex of mice in each group. n = 6, *p < 0.05, ns indicates p > 0.05. [file CPR-58-e70061-s004.jpg]

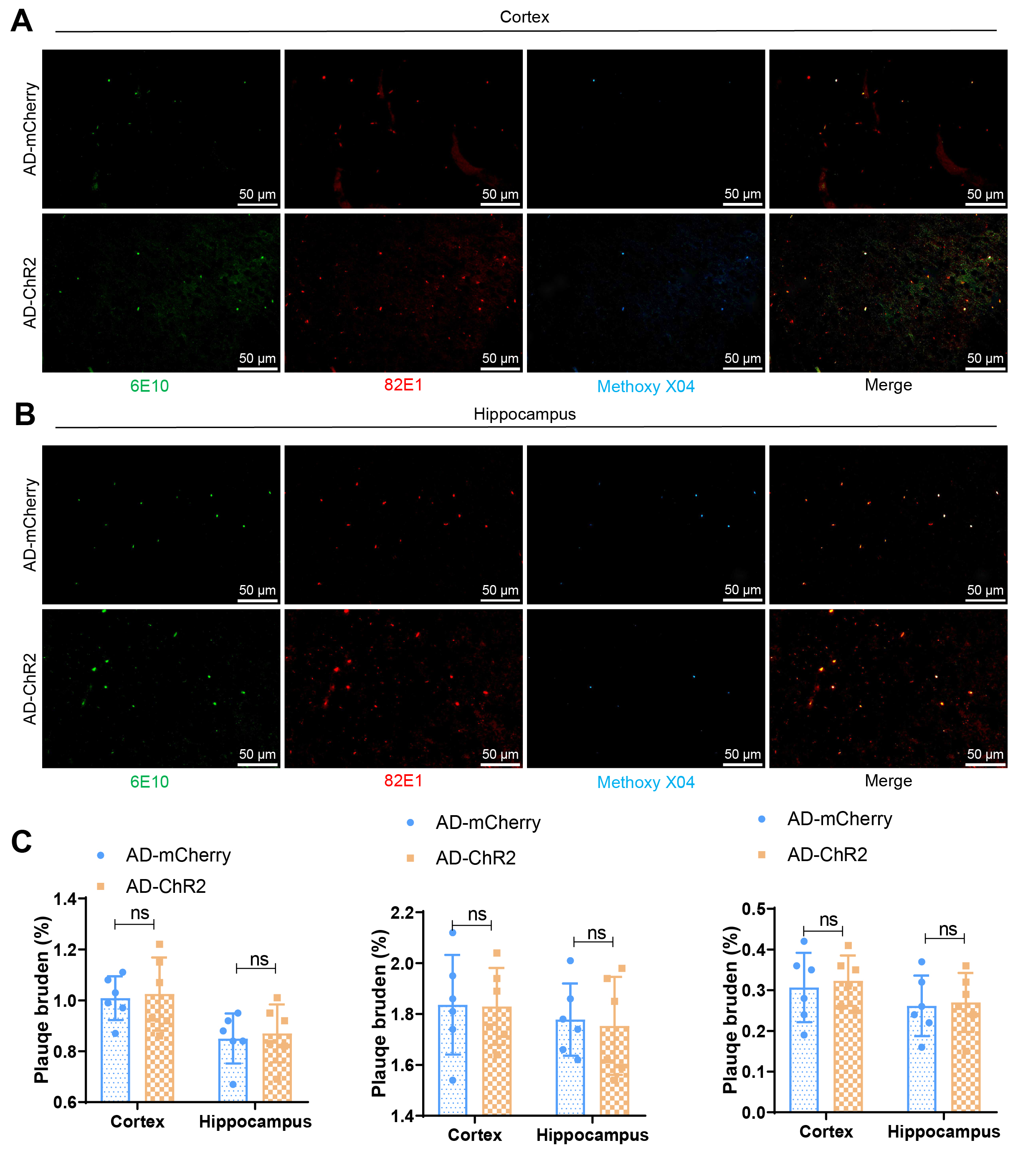

Supplement: Supplementary file 6 — Figure S6. Impact of isolated ChR2 expression on plaque deposition in AD mouse models. (A–C) Typical images and statistical data of 6E10 (green), 82E1 (red) and Methoxy‐XO4 (blue) positive amyloid plaques in the cortex (A) and hippocampus (B), scale bar = 50 μm. n = 6, ns indicates p > 0.05. [file CPR-58-e70061-s007.jpg]

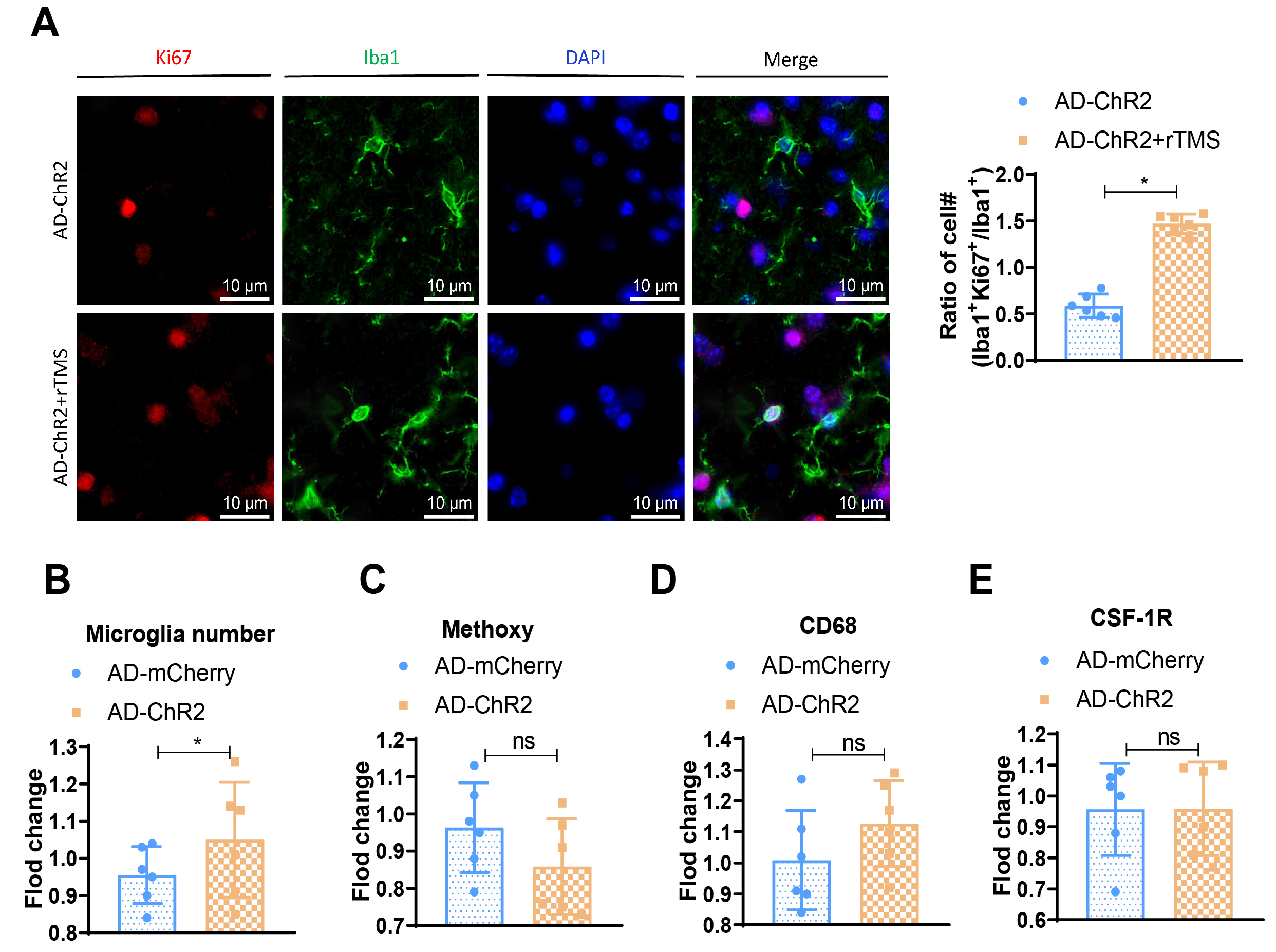

Supplement: Supplementary file 7 — Figure S7. Functional impact of rTMS stimulation on microglia in AD mice. (A) Representative images of Ki67 (red), Iba1 (green) and DAPI (blue) immunostaining, scale bar = 10 μm; (B, C) quantification of Aβ‐phagocytic microglia (Methoxy‐XO4+ microglia), quantitative analysis of the microglial population (B) and Methoxy‐XO4+CD11b+CD45low microglial cells (C); (D, E) quantification of microglial CD68 (D) and CSF‐1R (E) expression levels. n = 6, *p < 0.05, ns indicates p > 0.05. [file CPR-58-e70061-s006.jpg]

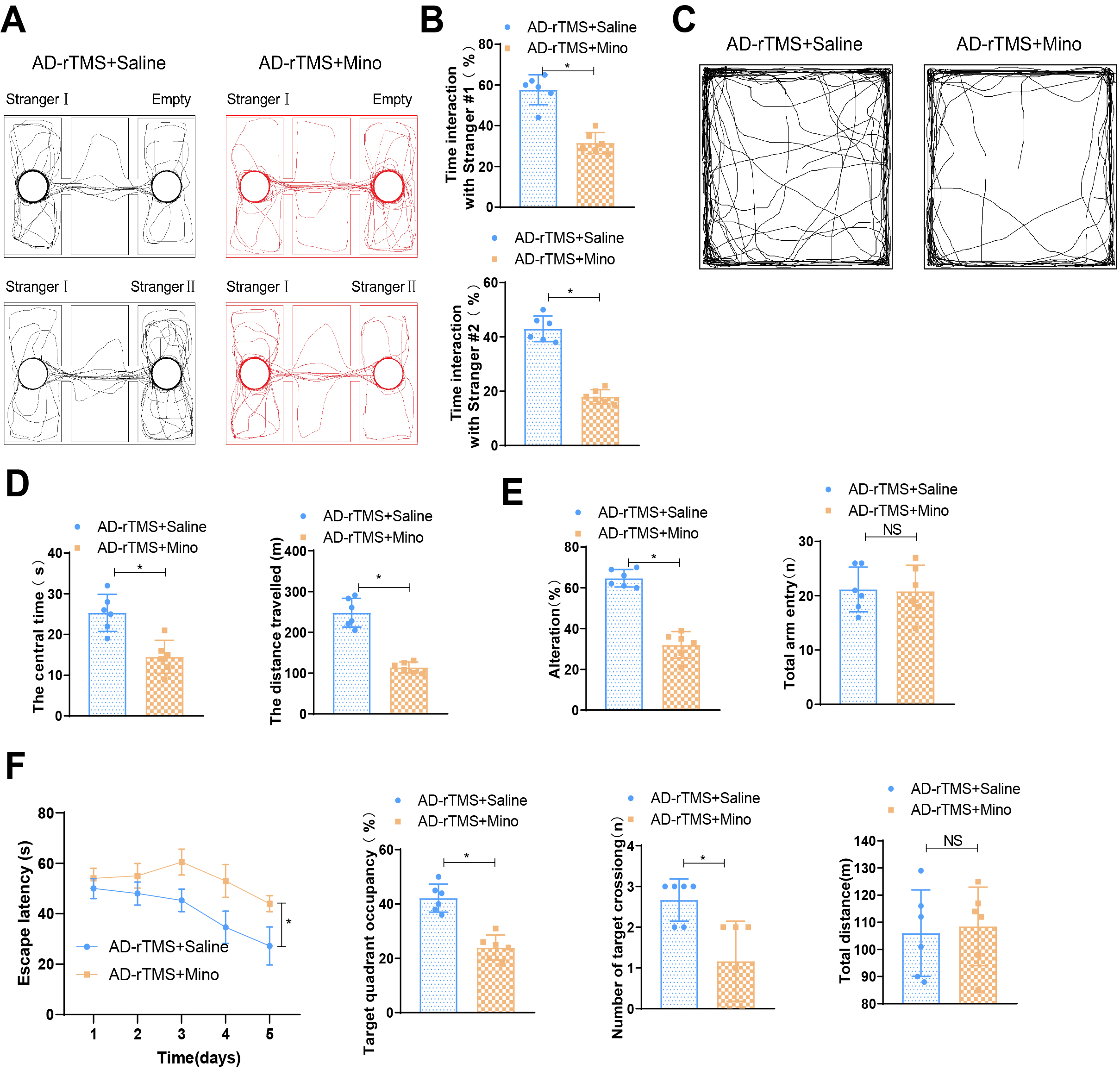

Supplement: Supplementary file 8 — Figure S8. rTMS stimulation delays AD progression depending on activated microglia. (A) Track plots of mouse behaviour in the three‐chamber social behaviour test; (B) bar graph presenting the duration of stay in each chamber during the three‐chamber social behaviour test for different groups of mice; (C) track plots of mouse behaviour in the open field test; (D) bar graph showing the time spent in the center of the arena and total distance travelled by mice; (E) bar graph displaying the percentage of alternation and total arm entries in the Y‐maze test for different groups of mice; (F) bar graphs illustrating the escape latency, target quadrant occupancy, target crossings and total movement distance in the Morris water maze test. n = 6, *p < 0.05, ns indicates p > 0.05. [file CPR-58-e70061-s008.jpg]

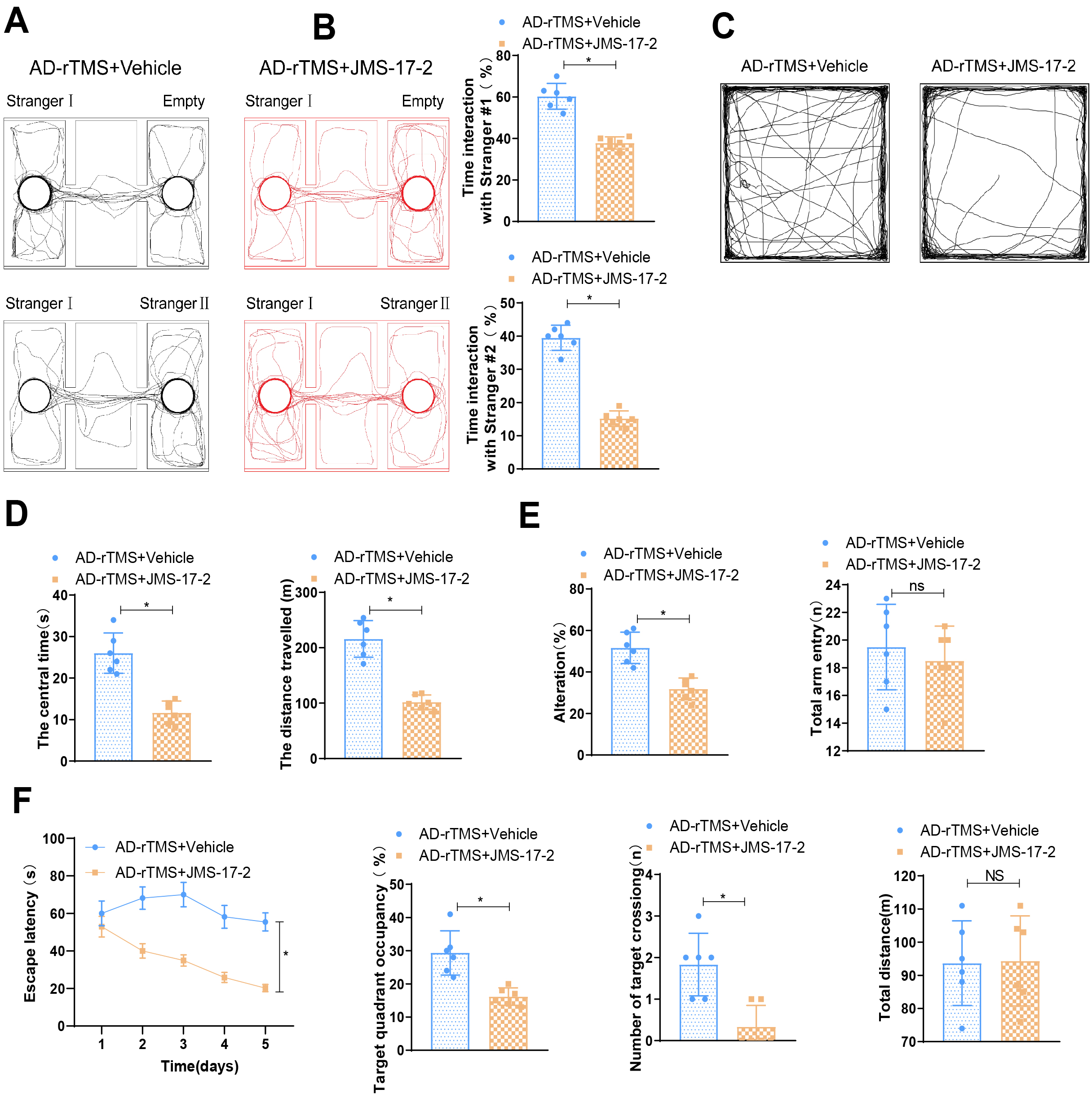

Supplement: Supplementary file 9 — Figure S9. JMS‐17‐2 impact on rTMS stimulation delaying AD. (A) Track plots of mouse behaviour in the three‐chamber social behaviour test for different groups of mice; (B) bar graph of the duration of stay in each chamber during the three‐chamber social behaviour test; (C) track plots of mouse behaviour in the open field test; (D) bar graph showing the time spent in the center of the arena and total distance travelled by mice; (E) bar graph displaying the percentage of alternation and total arm entries in the Y‐maze test; (F) bar graphs illustrating the escape latency, target quadrant occupancy, target crossings and total movement distance in the Morris water maze test. n = 6, *p < 0.05, ns indicates p > 0.05. [file CPR-58-e70061-s003.jpg]
