# Supplementary material for: Repetitive Transcranial Magnetic Stimulation Induces Cognitive Recovery in Alzheimer's Disease via GABAergic Neuron Activation of the Cx3cl1‐Cx3cr1 Axis
Source: Cell Prolif. 2025 May 25;58(12):e70061. doi: 10.1111/cpr.70061 (PMC12686124; doi:10.1111/cpr.70061)
Supplement: Supplementary file 10 — Table S1. RT‐qPCR primer sequence. [file CPR-58-e70061-s009.docx]

**Table S1. RT-qPCR primer sequence**

| **Gene** | **Sequences** |
| --- | --- |
| Cx3cl1 (house mouse) | Forward: 5’-CTGGCCGCGTTCTTCCATT-3’  Reverse: 5’-GCACATGATTTCGCATTTCGT-3’ |
| Tnf-α (house mouse) | Forward: 5’-ACCCTCACACTCACAAACCAC-3’  Reverse: 5’-ACAAGGTACAACCCATCGGC-3’ |
| Il-1b (house mouse) | Forward: 5’-GCCACCTTTTGACAGTGATGAG-3’  Reverse: 5’-GACAGCCCAGGTCAAAGGTT-3’ |
| Il-6 (house mouse) | Forward: 5’-TTCCTCTGGTCTTCTGGAGT-3’  Reverse: 5’-TGACTCCAGCTTATCTCTTGGTT-3’ |
| Gapdh (house mouse) | Forword: 5'- GTGGCAAAGTGGAGATTGTTG -3' |
|  | Reverse: 5'- CGTTGAATTTGCCGTGAGTG -3' |
